# A humanized mouse model for *in vivo* evaluation of invariant Natural Killer T cell responses

**DOI:** 10.3389/fimmu.2022.1011209

**Published:** 2022-10-03

**Authors:** Noemi Alejandra Saavedra-Avila, Paolo Dellabona, Giulia Casorati, Natacha Veerapen, Gurdyal S. Besra, Amy R. Howell, Steven A. Porcelli

**Affiliations:** ^1^ Department of Microbiology and Immunology, Albert Einstein College of Medicine, Bronx, NY, United States; ^2^ Experimental Immunology Unit, Division of Immunology, Transplantation and Infectious Diseases, San Raffaele Scientific Institute, Milano, Italy; ^3^ School of Biosciences, University of Birmingham, Birmingham, United Kingdom; ^4^ Department of Chemistry, University of Connecticut, Storrs, CT, United States; ^5^ Department of Medicine, Albert Einstein College of Medicine, Bronx, NY, United States

**Keywords:** CD1d, iNKT cell, Alpha-GalCer, transgenic mice, tumor immunity, humanized mouse models

## Abstract

Invariant natural killer T (iNKT) cells mediate immune responses when stimulated by glycolipid agonists presented by CD1d. In extensive studies of synthetic analogues of α-galactosyl ceramides, we identified numerous examples of significant differences in the recognition of specific glycolipids in wild type mice versus human iNKT cell clones or PBMC samples. To predict human iNKT cell responses more accurately in a mouse model, we derived a mouse line in which compound genetic modifications were used to express a human-like iNKT cell TCR along with human CD1d in place of the endogenous mouse proteins. Detailed transcriptional and phenotypic profiling demonstrated that these partially humanized mice developed an expanded population of T cells recognizing CD1d-presented glycolipid antigens, among which a subset characterized by expression of chemokine receptor CXCR6 had features characteristic of authentic iNKT cells. Responses to iNKT cell activating glycolipids in these mice generated cytokine production *in vitro* and *in vivo* that showed a pattern of fine specificity that closely resembled that of cultured human iNKT cell clones. Anti-tumor responses to variants of α-galactosyl ceramide in VαKI mice also correlated with their potency for stimulating human iNKT cells. This genetically modified mouse line provides a practical model for human presentation and recognition of iNKT cell activators in the context of a normally functioning immune system, and may furnish valuable opportunities for preclinical evaluation of iNKT cell-based therapies.

## Introduction

Invariant Natural Killer T cells (iNKT cells), also known as Type I NKT cells, are a conserved subset of specialized T cells that contribute to many innate and adaptive immune responses (1, 2). Unlike conventional T cells, iNKT cells express T cell antigen receptors (TCRs) of limited diversity, and respond mainly to specific foreign and self-glycolipid antigens presented by the MHC class I-like CD1d protein (3). The activities attributable to iNKT cells in a range of well-defined mouse models include tumor immunosurveillance and induction of tumor regression (4–6), regulation of autoimmunity and inflammation (7) and control of a variety of infections (8). Cumulatively, these findings strongly imply a potential role for iNKT cell-directed therapies in cancer and a variety of other clinical applications (9, 10). Substantial efforts have been directed at the development of synthetic glycolipid antigens that activate iNKT cells, and their use as potential therapeutics in strategies to capture the remarkable anti-cancer and adjuvant activities of iNKT cells (11).

The best-characterized glycolipid antigens recognized by iNKT cells are synthetic forms of α-galactosylceramides (αGalCer), which rapidly and potently activate the majority of iNKT cells in mice and humans to stimulate their proliferation, cytokine production and cytotoxic functions (12). The prototypical form of αGalCer, known as KRN7000, has been extensively studied for its therapeutic effects in mouse models of cancer, and has progressed into early phase human clinical trials in subjects with various cancers (13–15). While KRN7000 has been shown to stimulate immune activation *in vivo* in humans, anti-cancer effects have not been consistently observed in clinical studies to date (13, 14). Ongoing efforts seek to develop more potent iNKT cell activators for targeting human cancer, and for other applications in humans such as the design of vaccine adjuvants and immunotherapies for a variety of diseases (16, 17). Screening and optimization of numerous synthetic derivatives of αGalCer have relied mainly on the use of standard laboratory mice, especially with regard to *in vivo* testing. While most general features of the CD1d presentation of glycolipids are conserved between mice and humans, subtle differences exist that may result in inaccurate and misleading conclusions when extrapolating from one species to the other.

To create a more accurate system for *in vivo* assessment of iNKT cell directed therapies, several approaches have been used to generate mouse models with humanized iNKT cell responses. One approach has been to reconstitute a human immune system by transplanting hematopoietic stem cells into mice lacking endogenous lymphocytes (18–20). A second and probably more practical approach has been to carry out direct modifications of the mouse genome to humanize key elements in the CD1d-iNKT cell interaction while preserving an otherwise undisturbed mouse immune system. A significant first step toward the latter approach was taken by Yuan and colleagues with the generation of human CD1d knock-in mice (hCD1dKI), in which the coding regions of the mouse CD1D gene are replaced with the homologous human sequences (21, 22). In a second generation of this model, the hCD1dKI mice were further modified by a combined transgenic and gene knockout approach to replace the mouse iNKT cell invariant TCRα chain with the orthologous human Vα24-Jα18 TCRα sequence (*TRAV10-TRAJ18*) modified to contain a mouse TCRα constant domain for efficient pairing with endogenous murine TCRβ chains (23, 24). An initial publication in 2015 on these compound transgenic/knock-in mice showed that they developed functional iNKT cells and partially characterized their phenotypes and functions (24), but subsequent follow up studies have not yet appeared.

In the current study, we have re-constructed this compound knock-in and transgenic model of the humanized iNKT cell-CD1d axis, including an improved targeted deletion of the mouse Jα18 locus (*Traj18*) to specifically eliminate expression of endogenous mouse iNKT cell invariant TCRα chains without distorting the overall conventional T cell repertoire (25). Our extensive analysis showed that this stable homozygous mouse line, which we designated “VαKI”, developed functional iNKT cells while maintaining a complete and normally functioning mouse immune system, including an undisturbed and highly diverse conventional T cell repertoire. Similar to what has been described for hCD1dKI mice, the VαKI animals showed levels of iNKT cells that were readily detectable in most tissues, although reduced to levels that were more comparable to normal humans than to wild type mice. Importantly, the VαKI mice responded robustly to iNKT cell activators with a pattern of fine specificity that closely resembled cultured human iNKT cell clones. Our results support the view that the VαKI mouse model provides a useful and practical small animal system for assessing iNKT cell directed strategies for downstream translational work.

## Materials and methods

### Ethics statement

Animal experiments were conducted in accordance with the Policy on Humane Care and Use of Laboratory Animals of the United States Public Health Service. The protocol for vertebrate animal research in this study was approved by the Institutional Animal Care and Use Committee at the Albert Einstein College of Medicine (Animal Welfare Assurance Number D16-00200). Carbon dioxide inhalation was used for euthanasia, and all efforts were made to minimize animal distress, pain and suffering. This study did not involve the recruitment or participation of human subjects.

### Mice

Wild type mice (C57BL/6J strain) were purchased from Jackson Laboratory. The human CD1d-KI mice were obtained from Dr. Weiming Yuan (University of Southern California), and were bred and maintained in the animal facilities of the Albert Einstein College of Medicine. Production of transgenic mice expressing the human Vα24-Jα18 TCRα chain on the C57BL/6 background has been described, and the TCR transgenic line used for the current study was the Vα24^low^ line reported to express the transgene on ~20% of circulating lymphocytes (23). The Jα18-/- mice, also on the C57BL/6 background, were obtained from Dr. Mitchell Kronenberg (La Jolla Institute for Allergy and Immunology) (25). Animals were maintained under specific pathogen free conditions according to the guidelines of the Association for Assessment and Accreditation of Laboratory Animal Care. For experiments, animals were used at 6-10 weeks of age. Characterization of cell populations in mouse tissues was done using male mice. The iNKT activation studies *in vitro* and *in vivo* as well as tumor immunotherapy experiments were performed in female mice.

### Genotyping

Tail fragments were removed with a scalpel and digested overnight at 55°C in a 50 μL volume of 0.5 mg/mL solution of proteinase K (Qiagen, Germany) with 0.5% Triton-X in Gittschier buffer (26). Digested samples were diluted 10 fold with ultrapure water and were heated to 85°C for 5 min to inactivate proteinase K. Standard PCR reactions were performed with the following primers: mCD1d (Forward ATA TTT GAG GCA GGC TGT ACC AGC TGA AAT; Reverse GAA GCC AGA GAC ATG ACA CAC CAG CTG CCT; amplicon 343 bp), hCD1d (Forward CCT GGG ACC AAG GCT TCA GAG; Reverse CCT GCT GTT TCT GCT GCT CTG; amplicon 504 bp), Vα24 (Forward CTG GAT GCA GAC ACA AAG CAA AGC; Reverse GGA TCC TCA ACT GGA CCA CAG CCT CAG; amplicon 600 bp), Jα18 (Forward GAG GTT GAA CAA AGG AAG TGG; Reverse CCA CAA ATG GTA GTC AGT AGG; amplicon for KO 353 bp, amplicon WT 1083 bp). Offspring consistent with homozygosity at all loci were observed after more than three generations of sibling matings (i.e., hCD1d was expressed without expression of mCD1; Jα18 locus was absent and all mice were positive for Vα24 transgene expression).

### Generation of BMDCs

Mouse bone marrow-derived DCs were generated using bone marrow suspensions from 6–8 week old C57BL/6 mice, hCD1dKI and VαKI according to a published protocol (27). Bone marrow cells were harvested from femurs and red blood cells were depleted. Cells were resuspended in a medium consisting of RPMI-1640 supplemented with 10% heat-inactivated fetal bovine serum (FBS), 10 ng/ml mGM-CSF, and 50 mM 2-mercaptoethanol, 100 IU/ml penicillin, and 100 *μ*g/ml streptomycin and cultured (37°C, 5% CO_2_) in 10 cm Petri dishes at 1 × 10^6^ cells. On days 3, 5 and 7 of culture fresh mGM-CSF was added, and on day 10 the cells were collected.

### Synthetic glycolipid antigens

The glycolipid antigens used in this study were synthesized and characterized as previously described (28–30). The α-C-GalCer (C-glycoside) was obtained from the NIH Tetramer Core Facility (31). For *in vitro* assays, glycolipid stock solutions were prepared at 100 mM in DMSO (Sigma). Immediately before use, these stocks were heated to 70°C, sonicated for 5 min and then diluted to 1 mM in pre-warmed (37°C) culture medium (RPMI-1640 with 10% FCS). This stock was further diluted with culture medium immediately before adding to cell cultures to give the desired final glycolipid concentrations ranging from 0.01 - 1000 nM and a final DMSO concentration of 1%. For *in vivo* injection into mice, glycolipids were first dissolved to 20 mM in DMSO and then further diluted to 200 μM using PBS + 0.5% Tween-20. This solution was diluted 1:10 with pre-warmed (80°C) PBS immediately before injection of mice. Injection of 0.2 ml per mouse *via* the retro-orbital plexus delivered 4 nmol of glycolipid intravenously in a vehicle with final composition of PBS + 0.1% DMSO + 0.05% Tween-20.

### Tissue processing

Mouse spleens and livers were aseptically removed and mechanically disrupted to generate cell suspensions in sterile PBS. Red blood cells were removed by treatment with RBC lysing solution (Sigma). Liver suspensions were washed in PBS to remove fat, and mononuclear cells were separated by 30% Percoll density gradient with collection of the cell pellet. Venous blood was collected by facial vein puncture into a solution of PBS with 3 mg/mL EDTA, followed by lysis of red blood cells. Bone marrow cells were extracted by flushing femurs with PBS, followed by passage through 70 μm nylon mesh and red blood lysis. Mesenteric lymph nodes were pooled from individual mice and processed similarly to bone marrow samples.

### Flow cytometry

Cell suspensions were aliquoted into wells of 96-well microtiter plates for antibody staining. Extracellular staining with monoclonal antibodies, CD1d tetramer reagents and live/dead viability dyes was performed at 4°C. For intracellular staining, the cells were first fixed and permeabilized, and then stained with monoclonal antibodies following the protocol of the FoxP3/Transcription Factor staining buffer kit (Tonbo). Anti-human monoclonal antibody conjugates used were the following: phycoerythrin (PE)-conjugated-anti-hCD1d (clone CD1d42); brilliant violet 421 (BV421)- and FITC-anti-CD3 (UCHT-1); redFluor710-anti-CD3 (OKT3); PE-Cy5-anti-CD8α (RPA-T8); PECy7-anti-CD11c (3.9); BV510-anti-CD14 (MφP-9); Pacific Blue (PB)-anti-CD15 (MMA); BUV-anti-CD19 (SJ25C1); BUV737-anti-CD56 (NCAM16.2); PE-Dazzle594-anti-Vα24Jα18 (6B11). Anti-mouse monoclonal antibody conjugates used were as follow: BUV395- or PE-anti-B220 (RA3-6B2); FITC-anti-CD1d (1B1); BUV563-anti-CD4 (GK1.5); PECy5-anti-CD8α (53-6.7); PE-CFS594-anti-CD11b (M1/70); AF700-anti-CD11c (N418); PECy7-anti-CD205 (205yekta); PE-anti- CXCR6 (SA051D1); BV650-anti-F4/80 (BM8); BV510-anti-FcϵR1a (MAR-1); BV750-anti-IFNɣ (XMG1.2); eFluor450-anti-MHCII (M5/114.15.2); BV605-anti-NK1.1 (PK136); PECy7-anti-PLZF (R17-809), BV421-, PerCP-Cy5.5- or PE-CFS594-anti-TCRβ (H57-597), FITC-anti-γδ TCR (GL3); PE-anti-Vβ2 (B20.6), PE-anti-Vβ7 (TR310), PE-anti-Vβ8.1/8.2 (KJ16-133.18), PE-anti-Vβ8.3 (1B3.3). The following mouse and human tetramer reagents were obtained from the NIH Tetramer Core Facility: BV421-, PE- or APC-conjugated mCD1d tetramers and APC- or PE-conjugated-hCD1d tetramers, both loaded with αGalCer glycolipid PBS-57; BV421-conjugated mouse MR-1 tetramers loaded with 5-(2-oxopropylideneamino)-6-D-ribitylaminouracil (5-OP-RU). Data were acquired using a 4-laser Cytek Aurora spectral flow cytometer and analyzed using FlowJo software.

### *In vitro* and *in vivo* activation of iNKT cells

Mouse iNKT hybridoma lines derived from C57BL*/*6 mice (DN3A4-1.2), or from VαKI mice (VαKI-18) were stimulated using standard conditions with mouse BMDCs as APCs, and supernatants were harvested after 24 h for determination of levels of IL-2 by capture ELISA (32). Cloned human iNKT cell lines HDE3 and HDA7 were derived from healthy blood donors as previously described (33, 34). These were co-cultured in 96 well plates at a density of 2 x 10^4^ cells/well with 2 x 10^4^ hCD1d-transfected HeLa cells or human primary monocyte-derived DCs in 100 μL of RPMI-1640 medium supplemented with 10% FBS in a 5% CO_2_ incubator at 37°C. Glycolipid antigens were added at concentrations ranging from 0.1 – 100 nM. Supernatants were harvested after 24 h of culture, and concentrations of human IFNγ were measured by capture ELISA as described (33, 35). For stimulation of primary iNKT cells in splenocyte cultures, 10^6^ spleen cells from C57BL/6, hCD1dKI or VαKI mice were cultured with indicated glycolipid concentrations and incubated for 72 h, and mouse IFNγ was measured by ELISA. For *in vivo* stimulation of iNKT cells, C57BL/6, hCD1dKI and VαKI mice were injected i.v. *via* the retro-orbital plexus with 4 nmol of glycolipids in vehicle consisting of PBS plus 0.1% DMSO and 0.05% Tween-20. Mice were bled 2 h and 24 h later, and serum samples were stored at -80°C until cytokine measurement by ELISA.

### Single cell analysis of transcriptome and TCR expression

Splenocytes from VαKI and WT C57BL/6 mice were stained with live/dead viability dye (Zombie NIR), anti-B220, anti-TCRβ and human CD1d tetramers loaded with the αGalCer analogue PBS57. High speed sorting was carried out using a FACSAria fluorescence activated cell sorter (BD Biosciences) to collect tetramer positive and negative populations from live TCRβ+ and B220 negative cells. The sorted cells were loaded into the chips of the Chromium™ Single Cell 5′ Gel Beads Kit, followed by generation of single cell Gel Bead-In Emulsions (GEMs) using the Chromium Controller instrument according to the manufacturer’s instructions (10X Genomics). GEMs were then subjected to library construction using the Chromium™ Single Cell 5′ Library Kit v1 (10X Genomics). As a first step, reverse transcription was performed, resulting in cDNA tagged with a cell-specific barcode and a unique molecular index (UMI) for each transcript. Fragments were then size selected using SPRI select magnetic beads (Beckman Coulter), and Illumina sequencing adapters were ligated to the size-selected fragments and re-purified using SPRI select magnetic beads. Finally, sample indexes were incorporated and amplified, followed by a double-sided size selection using SPRI select magnetic beads. The quality of the final library was assessed using an Agilent 2100 Bioanalyzer (Agilent Technologies). The samples were then sequenced using a NextSeq instrument with 150 cycle paired end chemistry (Illumina). To process the sequenced libraries, generation of FASTQ, gene expression and count matrix files and generation of cloupe files were carried out with Cell Ranger software (10X Genomics). For the V(D)J libraries and generation of vloupe files, Cell Ranger V(D)J was used. Through this system, filtered UMI expression matrices from each sample were generated. Raw expression data was obtained containing transcriptomes for a pool of cells sorted from the spleens of three WT mice (C57BL/6) and three VαKI mice. In accordance with published pipelines and quality control standards, abnormal cells in all datasets were filtered out based on their gene expression distribution, and analysis was carried out with Seurat software from Satija Lab (36).

### Melanoma lung metastasis model

The B16-F10 melanoma cell line was obtained from ATCC (passage 3), and the experimental metastasis assay was performed as previously described (37, 38). C57BL*/*6 wild type mice, hCD1dKI, or VαKI mice were injected i.v. by the tail vein with 5 x 10^5^ B16-F10 cells in 200 µL of PBS. After 3 days, 4 nmol of glycolipids were administered by single i.v. injection in 0.2 ml vehicle (PBS + 0.05% Tween-20 + 0.1% DMSO). Two weeks after challenge, mice were sacrificed, lungs removed, and the area of melanized nodules on the lung surface was calculated from digital photographs of the excised lungs using Image J software from NIH (https://imagej.nih.gov/ij/index.html). Results were expressed as percentage of total lung surface area covered by melanized tumor growth.

### Statistical analysis

Data are shown as mean values with error bars representing one standard error (SE). Statistical analyses were done using GraphPad Prism software. Data involving three or more groups and multiple comparisons were analyzed for overall significance using one-way ANOVA, and level of significance for pairwise comparisons of selected groups was calculated using the Tukey post-test. Data involving single comparisons of two groups were analyzed for significance using the Mann-Whitney test. Values of P < 0.05 were considered significant.

## Results

### Generation of the VαKI mouse strain

**Figure 1:**
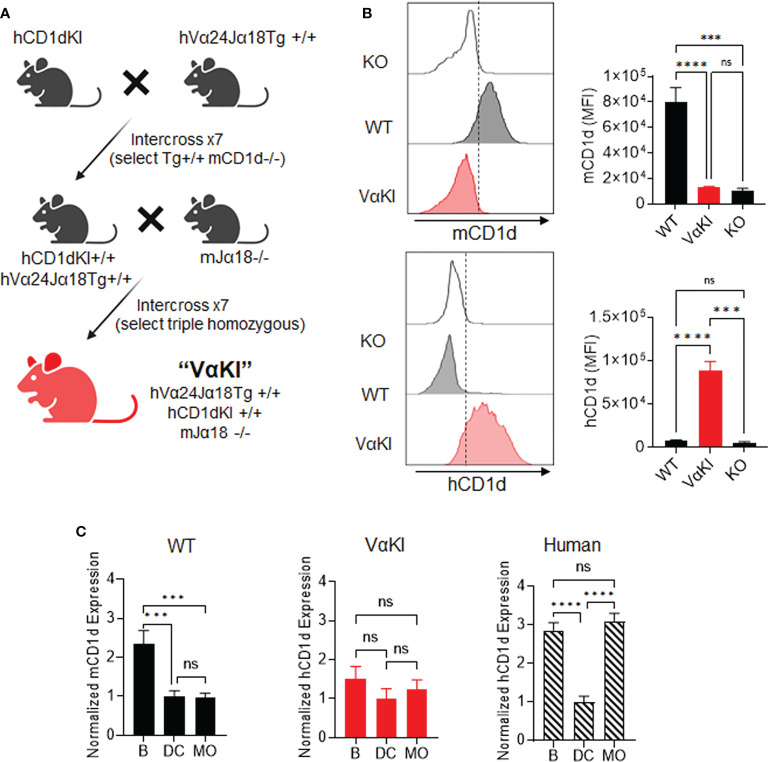
Generation of VαKI mice and their expression of human CD1d. **(A)** Schematic of breeding strategy to generate homozygous VαKI strain. All mice were C57BL/6 background. **(B)** Expression of mCD1d or hCD1d on BMDCs. Representative histograms are shown for flow cytometry gating on DEC205+ DCs (Zombie negative, CD11c+, MHCII+, CD8+, DEC205+) in cultured bone marrow cells from CD1D-/- (KO), C57BL/6 (WT) or VαKI mouse strains. The bar graphs show mean ± 1SE of MFI values for groups of 10 mice. **(C)** Expression of CD1d based on surface staining and FACS in PBMC from WT or VαKI mice (N = 15) or normal human blood donors (N = 10). Bars represent mean ± 1SE for mCD1d or hCD1d MFI values for B cells **(B)**, dendritic cells (DC) or monocytes (MO) with values normalized to levels on DCs (set at mean of 1). FACS gating strategy for leukocyte subsets is shown in **Supplemental Figures 1-3**. ***P < 0.001, ****P < 0.0001, ns, not significant (one-way ANOVA with Tukey post-test).

To generate a fully inbred mouse line with human CD1d and humanized iNKT cell TCR on the C57BL/6 background, we combined genetic modifications from three existing strains through multiple breeding and genotyping steps as illustrated schematically in **Figure 1A**. The human CD1d knock-in strain (22) was first crossed with mice carrying a homozygous transgene encoding a human Vα24-Jα18 (*TRAV10-TRAJ18* gene) cassette linked to the mouse TCRα chain constant region (hVα24Jα18Tg+/+) (23). Offspring were intercrossed and subsequent progeny with desired genotypes were selected to obtain animals homozygous for the knock-in and transgene loci. Although the TCR transgenic strain used in the initial cross also carried a deletion of the Jα18 gene to eliminate endogenous iNKT cell TCRs, we eliminated this particular allele during subsequent crosses since it was previously shown to cause a major distortion of the overall TCR repertoire (39). A second stage of breeding was used to reintroduce the Jα18 deletion using a more recently derived founder strain that maintained normal expression of other TCR genes (25). After selection of animals homozygous for all three modified loci, a stable breeding colony of this line, designated as VαKI mice, was established.

Analysis splenocytes of VαKI mice showed that all major leukocytes populations were present, with the total number and proportion of T cells similar to WT mice. Modest changes in numbers of B cells and all myeloid cell types were noted for VαKI compared to WT animals (**Supplemental Figure 1**). Further phenotypic analysis by flow cytometry confirmed the expected expression of human CD1d (hCD1d) and the absence of mouse CD1d (mCD1d) on leukocytes from VαKI mice (**Figure 1**; **Supplemental Figure 2**). Using cultured bone marrow-derived dendritic cells (BMDCs), known for their high expression of CD1d in wild type and hCD1dKI mice (22), we detected levels of hCD1d on the surface of BMDCs from VαKI mice that overlapped the levels for hCD1d on BMDC from hCD1dKI mice (**Figure 1B**). As expected, the VαKI BMDCs were completely negative for surface staining with antibodies specific for mCD1d. In addition to expression on cultured DCs, hCD1d was expressed at low but detectable levels on resting PBMCs of VαKI mice, with the highest surface levels on B cells, monocytes and DCs (**Supplemental Figure 2**). This pattern of hCD1d expression was similar to that observed for human circulating leukocyte subsets (**Figure 1C**; **Supplemental Figure 3**). Overall, hCD1d expression on most major leukocyte subsets in VαKI mice was detectable at a low level, and most prominently on B cells, monocytes and DCs, similar to the pattern observed for circulating cells in human blood (**Figure 1C**).

### Analysis of human Vα24 expressing T cells in VαKI mice

**Figure 2:**
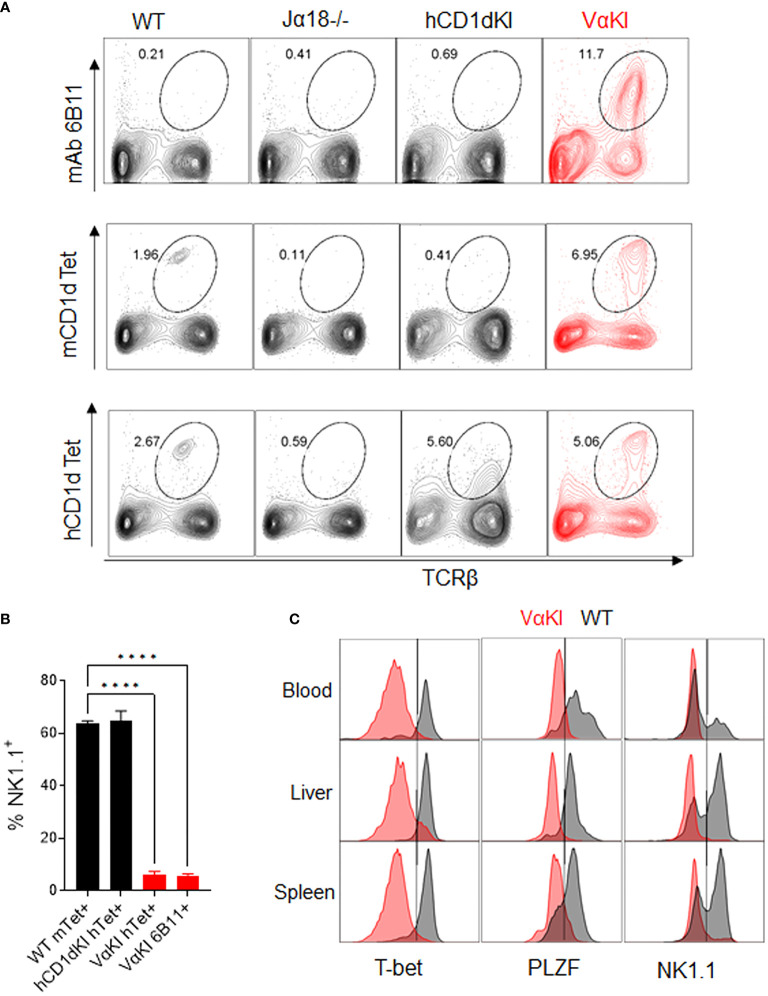
Vα24 transgene expression and CD1d tetramer binding of VαKI T cells. **(A)** Spleen cells from WT, Jα18-/-, hCD1dKI and VαKI mice were stained with mouse or human CD1d tetramers loaded with αGalCer glycolipid PBS-57, or with anti-human Vα24-Jα18 TCR specific mAb 6B11. Numbers in plots are percentages of total lymphocytes within the area enclosed by the ovals. **(B)** Percentages of NK1.1+ cells among gated CD1d tetramer + or 6B11+ cells in spleens of WT, hCD1dKI or VαKI mice. **(C)** Histograms of CD1d tetramer+ cells (Zombie negative, B220 negative, TCRβ+, CD1dTet+) showing their NK1.1, PLZF and T-bet expression in blood, liver, and spleen (VαKI mice shaded red and on WT C57BL/6 in black). Results shown are representative of three separate analyses. ****P < 0.0001 (one-way Anova with Tukey post test).

To detect expression of the humanized transgenic TCR on the surface of T cells in the VαKI mice, we used mAb 6B11 which is specific for an epitope formed by the in-frame rearrangement of human Vα24 to Jα18 (40, 41). Consistent with the published analysis of transgene expression in the hVα24-Jα18 Tg mice (23), splenocytes from VαKI mice showed a major population of T cells expressing the TCR transgene, comprising greater than 10% of all T cells. However, staining of splenocytes with either mCD1d or hCD1d fluorescent tetramers loaded with αGalCer identified four to five fold smaller populations (**Figure 2A**). Since binding of the tetramers indicates expression of a TCR capable of recognizing the complex of αGalCer bound to CD1d, this finding suggested that many of the 6B11+ cells in the VαKI animals were not iNKT cells. These most likely represented other types of T cells in which the hVα24 Tg was paired with endogenous TCRβ chains to generate conventional MHC-restricted T cells. We further investigated this possibility by using FACS to measure expression of the transcription factors T-bet and PLZF, and the cell surface receptor NK1.1 (CD161), which are markers expressed by the majority of canonical iNKT cells (42–45). This revealed that the majority of 6B11+ cells lacked expression of these markers (**Figure 2B**). Strikingly, most CD1d tetramer+ cells (>90%) in blood, spleen and liver of the VαKI mice also lacked expression of these markers, in contrast to the majority of tetramer+ cells in WT mice which expressed T-bet, PLZF and NK1.1 (**Figure 2C**).

**Figure 3:**
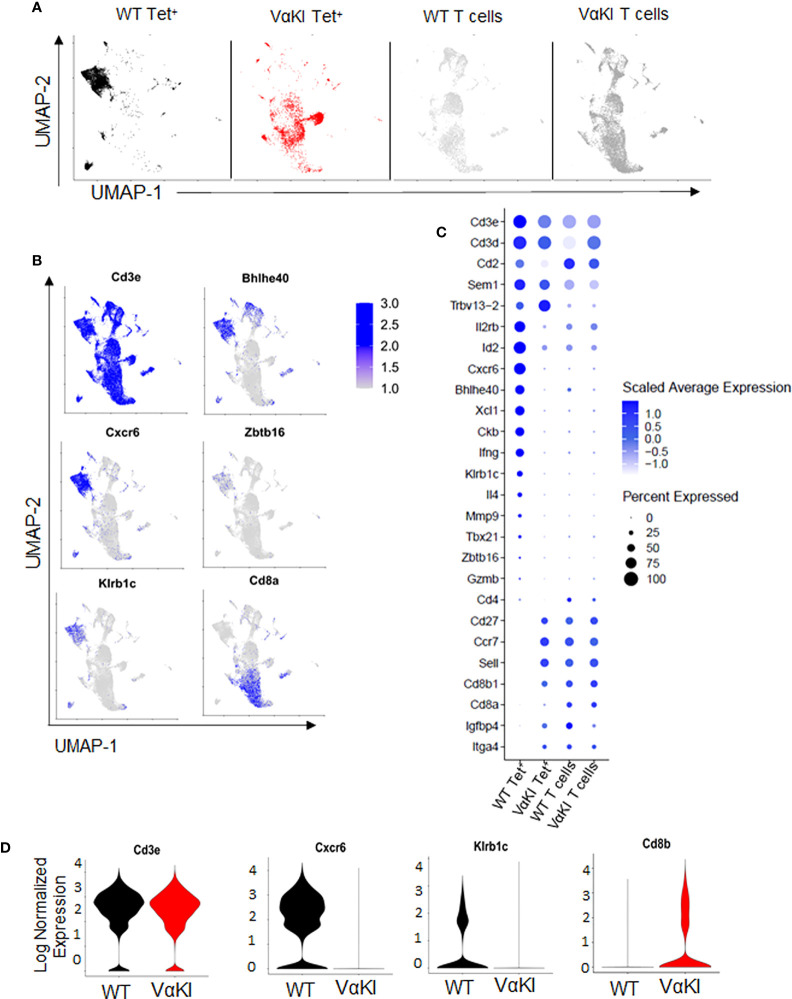
Gene expression profile of CD1d tetramer binding cells in VαKI mice. **(A)** UMAP plots showing analysis of single-cell mRNA sequencing data from spleen cells previously sorted for CD1d tetramer binding (Zombie negative, B220 negative, TCRβ+, CD1d Tet+) of C57BL/6 (WT) mice in black and VαKI in red. T cells not binding CD1d tetramers, representing predominantly conventional MHC-restricted T cells (Zombie negative, B220 negative, TCRβ+, CD1d Tet negative), are in gray). **(B)** mRNA expression of aggregated samples and their localization on the UMAP plots showing expression levels of selected genes with documented expression in WT mouse iNKT cells, including genes for CD3 epsilon subunit (Cd3e), CXCR6, NK1.1 (Kirb1c), CD8α, and transcription factors Bhlhe40 and PLZF (Zbtb16). Intensity of blue shading correlates positively with transcript expression level. **(C)** Bubble plots showing expression levels of transcripts for 26 genes selected for differential expression by Tet+ versus conventional (Tet negative) T cells. Blue shading indicates average expression level per cell, and size of symbols proportional to percentage of cells expressing detectable transcript levels. **(D)** Violin plots of mRNA expression of Cd3e, Cxcr6, Klrb1c and Cd8b in CD1d tetramer binding cells (Zombie and B220 negative, TCRβ+ and CD1d-Tet+) from WT (C57BL/6) and VαKI mice. All plots based on data from 4367 CD1d Tet+ cells from WT, 3403 Tet+ cells from VαKI, 6862 T cells from WT and 10998 T cells from VαKI.

To more deeply characterize the αGalCer-loaded CD1d tetramer binding cells in the VαKI mice, we carried out single cell transcriptome analysis of these cells purified from WT and VαKI spleens. Deep sequencing of the expressed transcriptomes revealed marked differences in global gene expression for the tetramer+ cells from the two mouse lines. When displayed as two-dimensional plots using uniform manifold approximation and projection (UMAP) dimensional reduction, the tetramer+ cells of VαKI mice showed little overlap with the clusters generated for WT tetramer+ cells (**Figure 3A**). Since the latter represent predominantly iNKT cells as defined in normal animals, these results suggested that most tetramer+ cells in VαKI mice may not be iNKT cells. In fact, at the transcriptome level, VαKI tetramer+ cells showed major overlap with several clusters defined in UMAP analysis carried out in parallel on unsorted T cells from either WT or VαKI animals., which gave similar patterns in both WT and VαKI mice (**Figure 3A**). Examination of the aggregated data confirmed exclusion of transcripts for several strongly iNKT cell associated genes from the VαKI tetramer+ cells, including *Cxcr6*, *Kirb1*c (NK1.1), *Bhlhe40* and *Zbtb16* (PLZF) (**Figure 3B**). In contrast, transcripts coding for CD8α and CD8β, which were absent in most clusters of WT tetramer+ cells, were present in a substantial fraction of VαKI tetramer+ cells (**Figure 3C**). Considering a panel of 26 genes known for their expression in iNKT cells, conventional MHC-restricted T cells or both, we observed a pattern of gene expression in the VαKI tetramer+ cells that was distinct from the tetramer+ cells of WT mice and more similar to unsorted T cells (**Figures 3C, D**).

**Figure 4:**
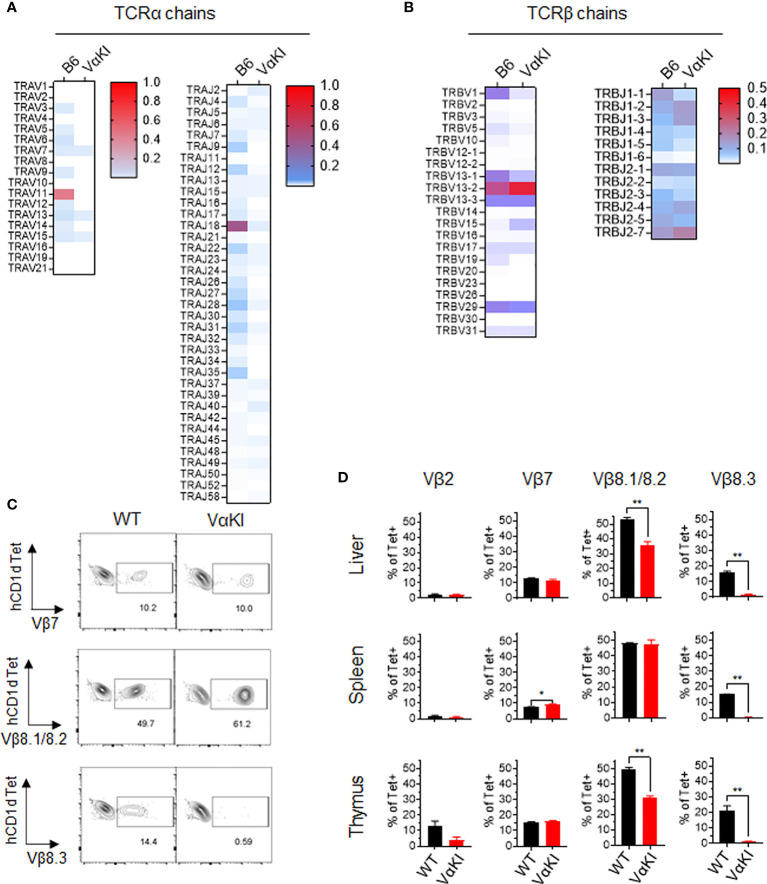
T cell receptor gene usage in CD1d tetramer binding cells from WT and VαKI mice. Sorted CD1d-Tet+ cells from WT (B6) or VαKI spleens were processed for single cell cDNA library construction, and paired TCRα and TCRβ chain sequences from 4352 WT and 3186 VαKI cells were obtained. **(A)** Heat maps for TCRα chain V and J gene segments. **(B)** Heat maps for TCRβ chain V and J gene segments. **(C)** Representative plots showing flow cytometry analysis of CD1d tetramer binding cells (B220 negative, TCRβ+ and CD1d-Tet+) in WT and VαKI mice using mAbs specific for the indicated TCR Vβ gene products. Number indicate the percentage of tetramer+ cells staining with each anti-Vβ specific antibody. **(D)** Bar graphs summarize the Vβ specific staining on tetramer binding cells in suspensions from spleen, liver and thymus for groups of WT (C57BL/6) or VαKI mice (N = 5 for each strain). Bars show mean ± 1SE. *P < 0.05, **P < 0.01 (Mann-Whitney test).

We also examined the TCR V and J gene expression of tetramer+ cells and tetramer negative T cells in VαKI compared to WT mice using single cell cDNA library sequencing. For purified tetramer+ cells, analysis of TCRα chain sequences showed the expected predominance of TRAV11 (i.e., coding for mouse Vα14) and TRAJ18 (coding for mouse Jα18). In contrast, the VαKI tetramer+ cells showed an absence of TRAV11-TRAJ18 and generally lower expression of mouse TCRα transcripts due to the dominant expression of the human Vα24-Jα18 transgene (**Figure 4A**). Examining TCRβ chain transcripts, we observed the expected predominance of TRBV1, TRBV13 and TRBV29 (encoding Vβ2, Vβ8 family and Vβ7, respectively). A substantially similar pattern of T cell receptor β chain gene usage was present in the VαKI tetramer+ cells, with cells from both strains showing similar diverse TRBJ (Jβ segment) gene usage (**Figure 4B**). Preferential expression of Vβ2, Vβ7 and especially Vβ8.1/8.2 was confirmed for both WT and VαKI tetramer+ cells at the surface protein level using specific mAb staining (**Figures 4C, D**). Taken together, these findings indicated that the majority of CD1dTet+ cells in the VαKI mice expressed a humanized TCR consisting of the transgenic human Vα24-Jα18 paired with endogenous mouse TCRβ chains similar to those generally used by normal mouse iNKT cells. An analysis carried out in parallel on TCR gene expression by tetramer negative T cells revealed highly diverse and substantially similar TCRα and TCRβ gene usage for both WT and VαKI mice (**Supplemental Figure 4**). This indicated that the T cell receptor repertoire of conventional MHC-restricted T cells was not significantly altered or distorted by the expression of the TCRα transgene in VαKI mice, which may be explained in part by the incomplete penetrance of expression of this particular transgene (23). We also used flow cytometry to assess the presence of TCRγδ expressing T cells and MR1-restricted MAIT cells, these being the two other well characterized MHC-unrestricted T cell populations of mice and humans. This showed these populations to be present in the spleens of VαKI mice at frequencies comparable to WT mice, whereas livers of VαKI mice showed a significant increase in TCRγδ+ cells and a moderate reduction in MAIT cells (**Supplemental Figure 5**).

### Identification of a subset analogous to iNKT cells in VαKI mice

**Figure 5:**
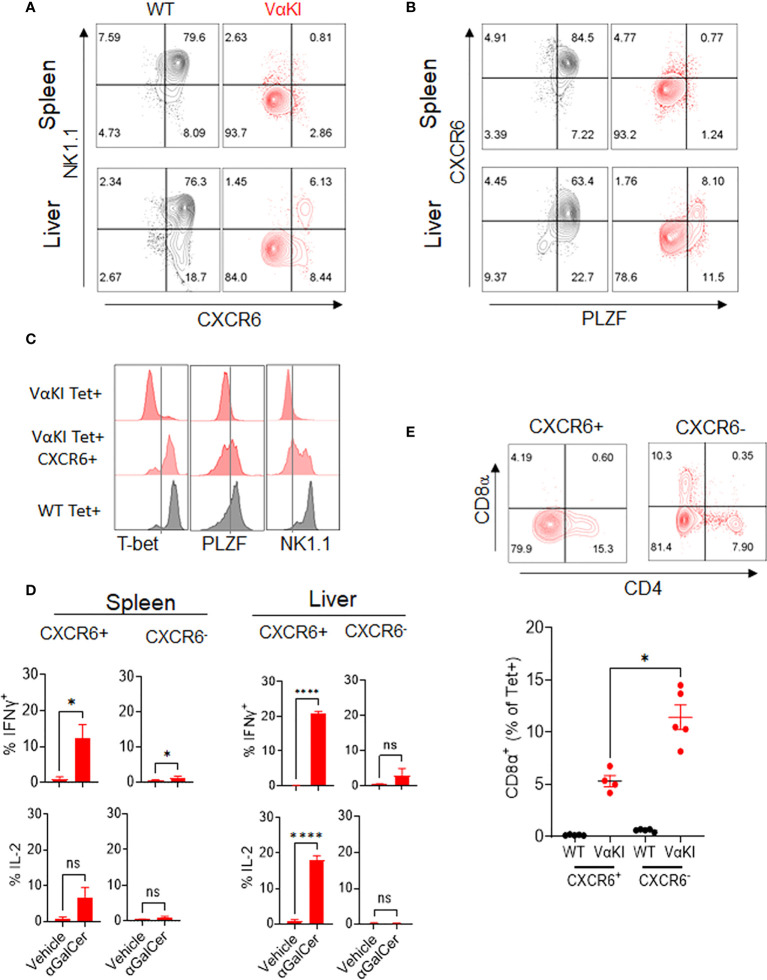
Identification of functional iNKT cells in VαKI mice based on CXCR6 expression. **(A)** NK1.1 and CXCR6 protein expression, and **(B)** PLZF and CXCR6 expression on CD1d tetramer+ cells in spleen and liver of WT (C57BL/6) and VαKI mice. Numbers in quadrants show percentage of total tetramer+ cells. **(C)** FACS analysis of intracellular T-bet and PLZF and cell surface NK1.1 on total or CXCR6+ CD1d tetramer+ cells from spleens of VαKI mice. Staining of total CD1d tetramer+ cells from WT mice is shown for comparison. **(D)** FACS analysis of staining for intracellular IFNɣ and IL-2 in CD1d-Tet+ cells from spleen or liver harvested 2 hours after i.v. injection of 4 nanomoles of αGalCer (glycolipid 7DW8-5) or inert vehicle. Staining with mAb to CXCR6 was included to separately analyze the CXCR6+ and CXCR6 negative subsets of tetramer binding cells. **(E)** FACS analysis of CD4 and CD8α expression in splenic CD1d-tetramer binding cells, gated on CXCR6 positive and negative subsets. Representative plots for one individual mouse among five analyzed are shown, and the graph below shows results for all animals with mean ± 1SE. ns, not significant *P < 0.05, ****P < 0.0001 (Mann-Whitney test).

While our analysis indicated that most of the expanded CD1dTet+ cells in the VαKI mice lacked the gene and protein expression profiles expected for canonical iNKT cells, a deeper analysis identified a subpopulation of cells that were phenotypically and functionally consistent with such a cell type. Using antibody staining for NK1.1 and chemokine receptor CXCR6, which our gene expression analysis showed to be highly enriched in iNKT cells of WT mice (**Figure 3**), we noted expression of one or both of these markers on a subset of the Tet+ cells in the VαKI animals (**Figure 5A**, and **Supplemental Figure 6A**). In addition to co-expressing NK1.1, a substantial fraction of the CXCR6+ Tet+ cells also had detectable levels of PLZF, which is highly associated with an iNKT cell program of differentiation (43) (**Figure 5B**). In thymus, the levels of PLZF in Tet+ cells varied from low to high, and this feature together with intracellular staining of transcription factor RORγT demonstrated distinct populations of Tet+ cells corresponding to previously described iNKT1, iNKT2 and iNKT17 subsets in VαKI mice (**Supplemental Figure 7**) (46). Functional analysis of responses to αGalCer stimulation also showed that the rapid cytokine production characteristic of iNKT cell function was observed in CXCR6+ Tet+ cells, but not in the more numerous CXCR6 negative Tet+ cells (**Figure 5D**). Based on these results, it was apparent that the CXCR6+ subset of the Tet+ population was most likely the functional equivalent of true iNKT cells in the VαKI mice. This conclusion was further supported by the analysis of CD4 and CD8 coreceptors on the CXCR6+ and CXCR6 negative Tet+ cells (**Figure 5E**; **Supplemental Figures 6B–D**). This showed that CD8α was excluded to a greater extent from CXCR6+ Tet+ cells compared to the CXCR6 negative population, which is also a characteristic of canonical iNKT cells (47). We also observed that a substantial fraction of the Tet+ CD8α+ cells in VαKI mice coexpressed CD8β, indicating the presence of CD8αβ heterodimers (**Supplemental Figure 6D**). This expression of CD8αβ was also observed on a small subset of Tet+ cells in WT mice, predominantly on the CXCR6 negative subset, although at much lower frequency than in VαKI animals.

**Figure 6:**
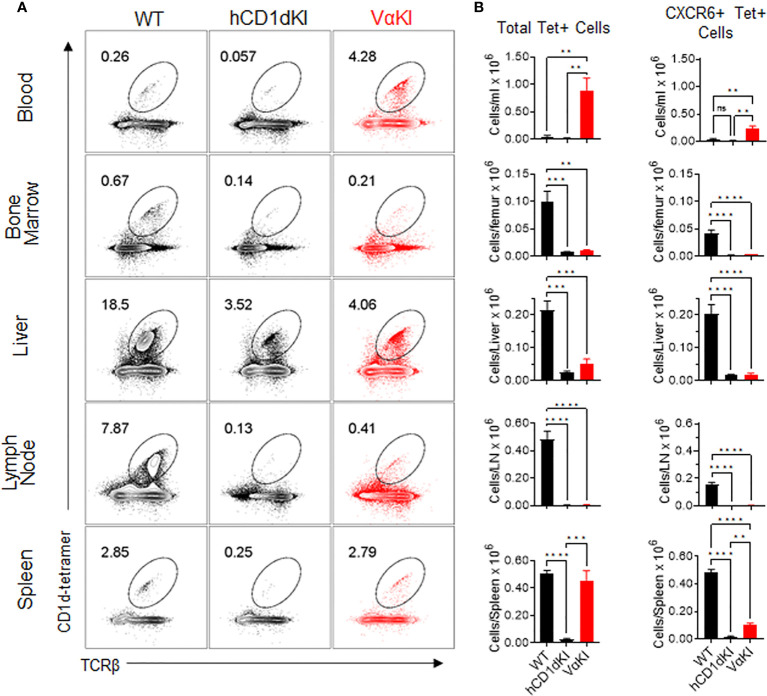
Frequency and numbers of iNKT cells in blood and various tissues of VαKI mice. **(A)** Representative dot plots from FACS analysis for CD1d-Tet and TCRβ staining of B220 negative live lymphocytes from each of the indicated tissues of WT (C57BL/6), hCD1dKI or VαKI mice. Lymph nodes were mesenteric nodes, and bone marrow was obtained from femurs. Numbers within the plots are the percentages of B220 negative live lymphocytes in the regions demarcated as ovals. **(B)** Bar graphs summarizing FACS results as in A for groups of 4 mice for each mouse strain. Absolute cell numbers were calculated based on total cell yields from each tissue, and bars show means ± 1SE. **P < 0.01, ***P < 0.001, ****P < 0.0001; ns, not significant (one-way ANOVA with Tukey post-test).

We used tetramer and CXCR6 staining to quantitate the levels of CD1d-αGalCer specific cells in suspensions from various tissues in VαKI mice, comparing these to levels in WT or hCD1dKI mice (**Figure 6**). For total Tet+ populations, VαKI mice showed uniquely high proportions and numbers of cells in the circulating peripheral blood, and relatively high levels similar to WT mice in the spleen. In other tissues examined (bone marrow, liver, lymph node), the levels of Tet+ cells were much reduced compared to WT, and resembled more closely the levels in hCD1dKI animals (**Figure 6B**). When focusing on only the CXCR6+ subset of the Tet+ cells, the levels in most tissues examined also showed a pattern indistinguishable from hCD1dKI mice, with the exception of modest elevations in blood and spleen. Overall, the functional iNKT cell population of VαKI animals, defined as Tet+ and CXCR6+ cells, showed a frequency that was much lower than in WT mice, and more typical for levels observed in humans as reflected in the previously described hCD1dKI mouse model (22).

### Glycolipid antigen recognition and antitumor responses in VαKI mice

**Figure 7:**
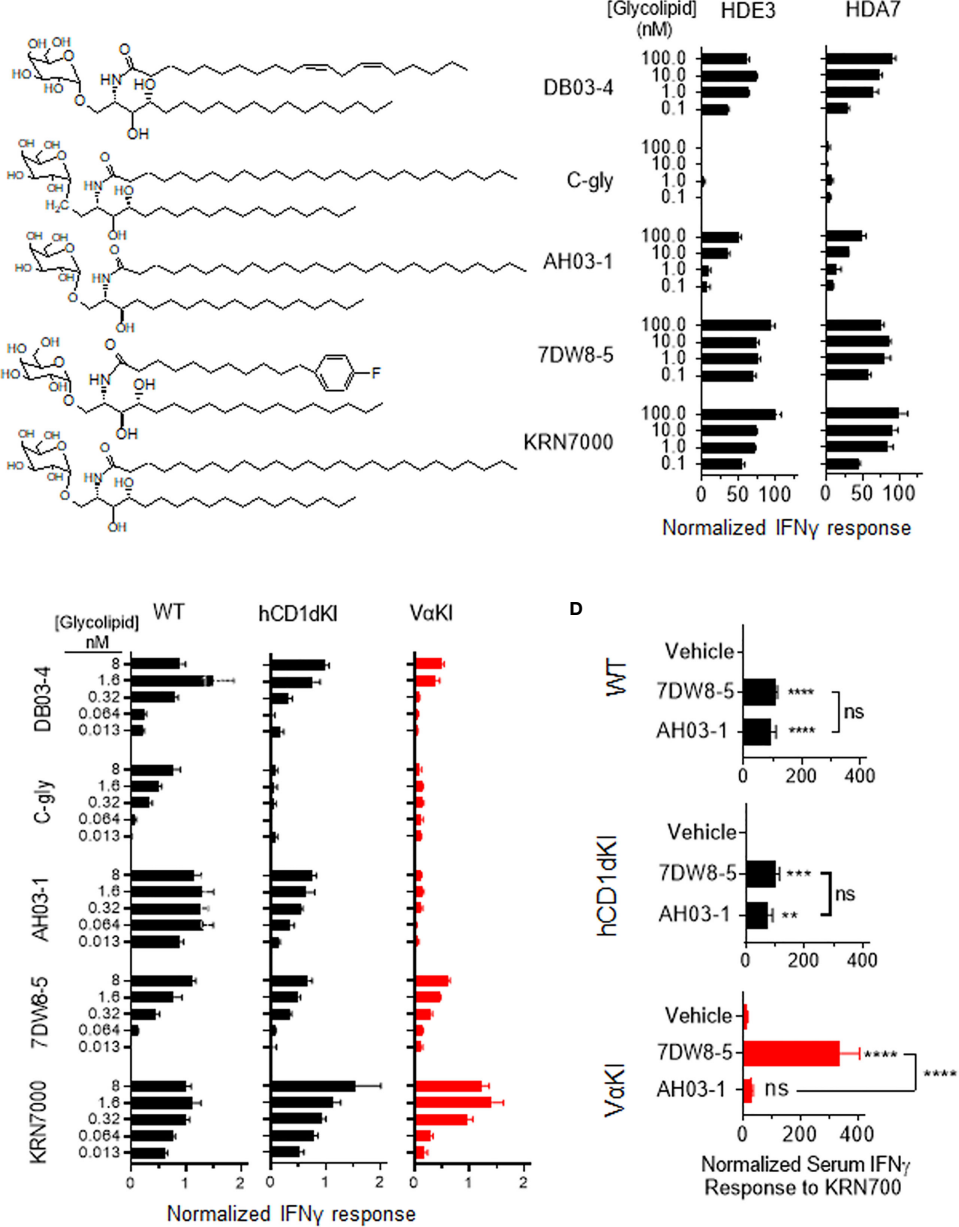
Human-like response of VαKI iNKT cells to αGalCer analogues. **(A)** Structures of glycolipids DB03-4, C-glycoside (C-gly), AH03-1, 7DW8-5 and KRN7000. **(B)** Human iNKT cell clones HDE3 and HDA7 were stimulated with different concentrations of glycolipids in cultures with hCD1d transfected HeLa cells as APCs. After 48 h, IFNγ concentrations in supernatants were determined by ELISA and normalized to levels induced by 100 nmol KRN7000. **(C)** One million spleen cells from WT, hCD1dKI or VαKI mice were stimulated in culture with the indicated concentrations of glycolipids. After 72 h, IFNγ concentration in the media was determined by ELISA and results were normalized to levels obtained with 40 nanomoles of KRN7000. **(D)** Four nanomoles of glycolipids (AH03-1 or 7DW8-5) or an equal volume of inert vehicle were administered i.v. into WT, hCD1d or VαKI mice. After 24 h, sera were collected and the concentration of IFNγ determined by ELISA. Asterisks in D indicate comparisons of glycolipids to vehicle, except for those associated with brackets which compare the two glycolipids to each other. **P < 0.01, ***P < 0.001, ****P < 0.0001, ns, no significant (one-way ANOVA with Tukey post-test).

To assess the fine specificity of glycolipid antigen recognition in VαKI mice, we used a panel of five well characterized synthetic forms of αGalCer that have been studied previously as potent iNKT cell activators in wild type mice (**Figure 7A**). These included the prototypical antigen KRN7000 (32) and the derivative 7DW8-5 with a modified fluorphenyl containing acyl chain (48). Both of these are known to stimulate human iNKT cells *in vitro* and have also been found to have detectable activities *in vivo* in human subjects (14, 49). Another acyl chain variant, DB03-4, has been described as a potent Th2 cytokine biasing iNKT cell activator in mice (28), and as a strong stimulator of human iNKT cells in cell culture (35). In addition, two variant glycolipids were included which are reported to stimulate pronounced Th1 cytokine biased iNKT cell dependent responses in wild type mice, namely the C-glycoside α-C-GalCer (C-gly) (31) and the 4-deoxy sphingosine derivative AH03-1 (29). Using previously established human iNKT cell clones co-cultured with hCD1d expressing APCs (33), we observed that these compounds varied in their potency based on levels of IFNγ released into culture supernatants (**Figure 7A**). Of particular note were the minimal or low responses of human iNKT cells to C-gly and AH03-1 in this context. The same panel of glycolipids was assessed for relative potency of iNKT cell stimulation by measuring IFNγ release in cultured spleen cells from WT, hCD1dKI or VαKI mice (**Figure 7B**). This revealed that the responses of VαKI cells showed a pattern that was more similar to that observed for human iNKT cell clones, with a particularly striking reduction in responses to C-gly and AH03-1 in comparison to WT mouse cells. This overall pattern was also evident with cultures of cells from hCD1dKI mice, except that the reduced response to AH03-1 was clearly more pronounced in the VαKI cultures (**Figure 7B**). In general, these trends were confirmed at the level of direct iNKT cell recognition using cloned iNKT cell hybridoma lines derived from WT versus VαKI mice (**Supplemental Figure 8**).

**Figure 8:**
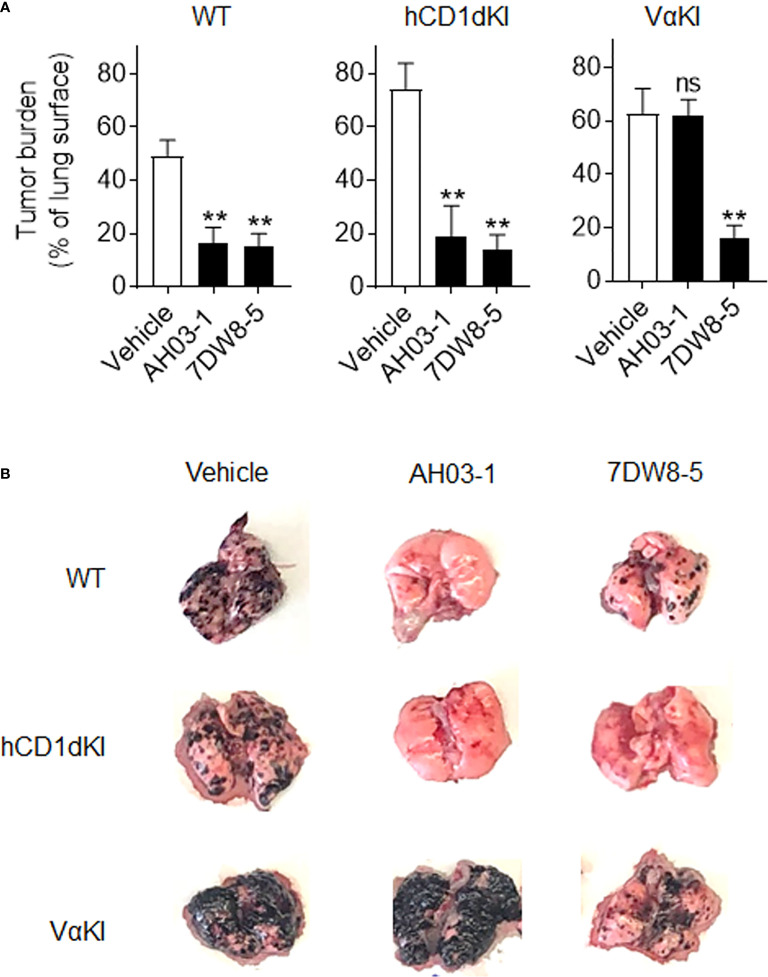
Anti-tumor activity and altered glycolipid responses in VαKI mice. **(A)** Groups of WT (C57BL/6), hCD1dKI or VαKI mice (N = 3 - 5 per group) received intravenous injection of 5 x 10^5^ B16-F10 melanoma cells on day 0. Three days later, glycolipids (4 nmol of AH03-1 or 7DW8-5) or inert vehicle were administered as treatment. Animals were sacrificed and lungs were extracted on day 15 and digital images obtained for analysis with ImageJ software to determine area of lung surface covered by melanized tumor. Bar graphs show mean percent of involved area ± 1SE. Asterisks indicate significant differences for glycolipid treated compared to vehicle treated groups. **P < 0.01, ns, not significant (one-way ANOVA and Tukey post-test for multiple comparisons). **(B)** Representative images of lungs an individual mouse from each group showing level of gross tumor involvement.

We also compared responses *in vivo* to αGalCer analogues in VαKI compared to WT or hCD1dKI mice. For this, we focused on the comparison of glycolipids AH03-1 and 7DW8-5, since these showed markedly different potencies for iNKT cell activation for WT mouse versus human iNKT cells in our *in vitro* analyses (**Figures 7A-C**). At 24 h after a single intravenous injection of either inert vehicle or glycolipids, animals were bled and serum levels of IFNγ were measured (**Figure 7D**). Wild type and hCD1dKI mice showed similar responses to both 7DW8-5 and AH03-1, indicating rapid iNKT cell activation *in vivo*. In contrast, VαKI mice responded strongly to 7DW8-5 but showed no significant response to AH03-1. This pattern was repeated in a test of anti-tumor activity of these glycolipids in the three mouse strains (**Figure 8**). Using the B16-F10 experimental lung metastasis model, which is well established to be responsive to treatment with KRN7000 or other forms of αGalCer (22, 24, 37, 38), we observed marked reductions in lung tumor burdens based on calculated areas of melanization on the lung surface following treatment with either 7DW8-5 or AH03-1 in both WT and hCD1dKI mice. In striking contrast, only 7DW8-5 was effective in VαKI mice, whereas AH03-1 showed no detectable effect on tumor burden. Thus, antitumor effects displayed a dependence on glycolipid ligand structure similar to that observed for iNKT cell responses *in vitro* and *in vivo*, with VαKI animals showing fine specificity for variant forms of αGalCer that mirrored that of fully human responses in cell culture.

## Discussion

The potential development of new approaches to immunotherapy involving CD1d-restricted iNKT cells will be greatly accelerated by accurate mouse models of human iNKT cell responses. Our approach to the generation of a practical mouse model for this application has used the incorporation of three stable genetic modifications to create a substantially humanized iNKT cell-CD1d axis on the background of an otherwise unmodified C57BL/6 mouse strain. The fully homozygous VαKI line that we characterized in the current study used the previously described human CD1d knock-in mice to introduce human CD1d expression while simultaneously eliminating the mouse CD1d coding sequence. The hCD1dKI mice express a fully human CD1d protein under the control of endogenous mouse regulatory elements, leading to a physiologically normal pattern of CD1d expression, regulation and function (22). By combining this with a human Vα24 transgene, we have effectively humanized the principle recognition component of the TCR of iNKT cells, which is the main determinant of the fine specificity of glycolipid antigen recognition (3).

Most previous efforts toward humanized mouse models for the study of iNKT cells have required the introduction of human hematopoietic stem cells into individual immunodeficient mice to populate these animals with human T cells and APCs. A pioneering study using this approach was first reported by Gumperz and colleagues, who showed that iNKT cells developed at detectable levels in NOD/Prkdc^scid^/γc^null^ (NSG) mice engrafted with human fetal thymus, liver and CD34^+^ hematopoietic stem cells, and could mount responses to αGalCer *in vivo* (18). Subsequent refinements of this approach were published by Tsuji and colleagues, who also incorporated adeno-associated virus delivery of multiple human cytokine genes and human CD1d to improve immune cell reconstitution and further humanize glycolipid antigen presentation (19, 50). While these models have been useful for demonstrating some basic principles of iNKT cell function *in vivo*, they suffer from many deficiencies with respect to incomplete development of a fully functional immune system and abnormal trafficking, growth and regulation of human cells within the mouse environment. In addition, such models are extremely costly and cumbersome to construct, and tend to suffer from poor reproducibility.

In contrast, the targeted genetic approaches used to generate the VαKI strain can produce robust and easily propagated mouse lines that are cost effective and yield highly reproducible experimental models. Previous applications using the simple transgenic approach generated mouse models that humanized either the CD1d protein or the TCRα chain as the dominant recognition element in the iNKT cell TCR (23, 51). These were useful first steps, but did not sufficiently humanize both the presentation and antigen recognition to capture the subtle interspecies differences and nuanced features of iNKT cell responses. The work by Yuan, Cresswell and colleagues leading to the generation of the hCD1dKI mice was an important incremental improvement, as these animals showed preservation of the native pattern of CD1d expression and also developed levels of iNKT cells that closely matched the levels observed in normal humans (22). These investigators subsequently carried the approach further by combining the hCD1dKI with a human Vα24 transgene, while also incorporating a deletion of the mouse Jα18 gene to eliminate the development of iNKT cells expressing a native mouse TCR, thus generating a more completely humanized transgenic model with respect to both antigen presentation and recognition (24).

To extend the important work by Yuan and colleagues, we have followed a very similar approach and essentially reconstructed their model to enable our analysis of this stable genetic system for studying human-like iNKT cell responses. There is however one major difference between our VαKI strain and the animals described previously, which is in the particular Jα18 knock out line used for the construction of the mice. In the case of the animals reported by Yuan’s group, a Jα18 deletion was used that contained a transcriptionally active neomycin resistance cassette, which causes a major reduction of TCR Vα diversity and a distortion of the TCR repertoire in the conventional MHC-restricted T cells of these mice (39). In constructing the VαKI strain, we instead used a more recently derived Jα18 knockout mouse in which the targeted deletion does not introduce other perturbations of the TCR repertoire (25). Our single cell sequencing of TCR transcripts confirmed that VαKI mice maintained a full repertoire of TCR diversity in both α and β chains of conventional T cells that closely resembled that of WT mice (**Supplemental Figure 4**).

Our analysis of the T cells of VαKI mice revealed strong but incomplete penetrance of the human Vα24 transgene expression, as expected from previous work on the relevant transgenic founder line (23). In addition, an expanded population of cells binding CD1d-αGalCer tetramers was readily detected in many tissues of these mice. Interestingly, a deeper analysis of these tetramer binding cells revealed that a majority of them were most likely not functional equivalents of canonical iNKT cells, as they lacked many of the key features of these cells at the levels of protein expression and transcriptional signatures. Nevertheless, a distinct population corresponding to functional iNKT cells could be identified in all tissues examined by staining for surface expression of CXCR6. This population of CXCR6+ tetramer binding cells was present in most tissues at levels similar to or slightly elevated compared to hCD1dKI mice (**Figure 6**), which has already been proposed to more closely approximate iNKT cell numbers in humans than what is found in WT mice (22). This CXCR6+ population in VαKI mice could be divided into iNKT1, iNKT2 and iNKT17 functionally distinct subsets (42, 46) (**Supplemental Figure 7**), and they responded rapidly to αGalCer stimulation *in vivo*, as opposed to the CXCR6 negative subset of tetramer binding cells which did not (**Figure 5C**).

The mechanism accounting for the reduced numbers of bona fide iNKT cells in VαKI compared to WT mice remains unknown, although a very similar phenomenon has been previously noted in human CD1d knock-in mice that have no direct manipulation of the TCR repertoire (22). Differences in intracellular trafficking to endosomal compartments of mouse versus human CD1d have been documented (52), and subtle disruptions of endosomal sorting of CD1d have been shown to influence the rate of positive thymic selection of iNKT cells (53). Thus, we speculate that alteration of intracellular trafficking of CD1d could lead to display of different endogenous lipid antigens, resulting in reduced efficiency of positive selection or increased rates of negative selection in the thymus of VαKI mice. Alternatively, the presence of different endogenous ligands for CD1d presentation in mouse and human cells could have an impact on positive or negative selection or peripheral expansion of iNKT cells to yield the observed reductions in iNKT cells in all tissues of VαKI mice that we examined except for blood (**Figure 6**).

With regard to the expanded CXCR6 negative tetramer binding cells in VαKI mice, it is unclear at present whether these represent a physiologically relevant population with distinct functions. These cells did not respond rapidly upon first exposure to αGalCer in the manner of canonical iNKT cells. However, the possibility that they may respond to this stimulus with delayed kinetics or only after repeated antigen exposure remains to be tested. While the presence of an expanded population of atypical tetramer binding cells may represent a limitation of the VαKI model in terms of accurate modeling of complex human immune responses or diseases, this may also provide opportunities to gain insight into the process of T cell development and the potential functions of rare T cell subsets that are not currently well studied in humans. For example, while the expansion of these atypical tetramer binding cells is likely an artifact of the forced expression of the transgenic TCRα chain, it is possible that they may correspond developmentally and functionally to the CD1d-αGalCer reactive T cells lacking canonical iNKT cell features that have been occasionally identified in cultures derived from normal human blood (54). Furthermore, a major conclusion proposed previously by Yuan’s study of their similar humanized mouse model was their demonstration of a CD8αβ+ iNKT cell subset, which in an adoptive transfer model showed anti-tumor activity (24). This phenotypic subset of iNKT cells has been reported in normal human PBMC at very low frequencies usually comprising less than 5% of circulating iNKT cells, but its role in immunity has not been established (41, 55). We also found that CD8αβ was prominently expressed among CD1dTet+ cells in VαKI mice (**Supplemental Figure 6**). However, these CD8αβ+ cells were predominantly contained within the CXCR6 negative fraction of CD1dTet+ cells, and thus their relationship to the canonical iNKT cells of the VαKI mice, which are mostly CXCR6+, remains unclear.

In summary, we propose that the VαKI mice described here have major practical advantages over the most notable previous efforts to produce humanized mice for the study of iNKT cell responses *in vivo*. Importantly, our results showed that subtle differences in structure of glycolipid ligands were detected by iNKT cells in VαKI mice in a manner that was distinct from WT mice, and highly suggestive of a more human-like pattern based on predictions from human cell culture studies. It remains to be determined if the iNKT cells of VαKI mice can mediate all of the many effector activities seen in normal non-transgenic cells, and the sensitivity of the VaKI iNKT cells to induction of anergy and costimulatory signals following TCR stimulation also will require further study. Nevertheless, our current results indicate that the VαKI animals should be useful for preclinical screening of iNKT cell activators, and will provide a valuable platform for rapid and cost-effective testing of new vaccines or immunotherapies that involve manipulation of iNKT cell responses. In addition, since the VαKI mice superimpose a potentially more human-like iNKT cell response onto a fundamentally normal mouse immune system, important cell interactions and other relevant factors should be preserved in these animals during the evolution of immune responses. This strongly implies that the use of VαKI mice for evaluating iNKT cell directed therapies in models of cancer or other diseases will offer advantages over standard mouse models for predicting outcomes in humans, thus accelerating progress in this area of translational science.

## Data availability statement

Transcriptomic data from this study have been deposited at the NCBI database. The following GEO Accession numbers can be found below: GSE213954 A humanized mouse model for in vivo evaluation of iNKT cell responses Sep 25, 2022 approved None; GSM6596844 B6 iNKT GE (14539) Sep 25, 2022 approved H5; GSM6596845 VAKI iNKT GE (14541) Sep 25, 2022 approved H5; GSM6596846 B6 T GE (15450) Sep 25, 2022 approved H5; GSM6596847 VAKI T GE (14542) Sep 25, 2022 approved H5; GSM6596848 B6 iNKT VDJ (14535) Sep 25, 2022 approved VLOUPE; GSM6596849 VAKI iNKT VDJ (14537) Sep 25, 2022 approved VLOUPE; GSM6596850 B6 T VDJ (14536) Sep 25, 2022 approved VLOUPE; GSM6596851 VAKI T VDJ (14538) Sep 25, 2022 approved VLOUPE.

## Ethics statement

The animal study was reviewed and approved by The protocol for vertebrate animal research in this study was approved by the Institutional Animal Care and Use Committee at the Albert Einstein College of Medicine (Animal Welfare Assurance Number D16-00200).

## Author contributions

NAS-A performed the experiments, NS-A and SP designed the experiments, NS-A and SP wrote the paper. AH, NV and GB designed and synthesized synthetic glycolipids. GC and PD provided transgenic mice and assisted in model design. All authors read and approved the manuscript.

## Acknowledgments

This work was supported by NIH grants R01 AI45889 (SP and NS-A), R01 GM111849 (AH, SP and NS-A), and Einstein Liver Center grant DK041296-31 (SP and NS-A). GB acknowledges support from a Personal Research Chair from Mr. James Bardrick and a Royal Society Wolfson Research Merit AwardGC was funded by Associazione Italiana Ricerca sul Cancro (AIRC) project grant IG2017-ID.20081. Core facilities used in this study (Einstein Flow Cytometry, Genomics and Bioinformatics Core Facilities) were supported in part by the Montefiore-Einstein Cancer Center (NIH/NCI grant P30CA13330), and used instrumentation obtained with funding from NIH Shared Instrumentation Grants 1S10OD019961-01, 1S10OD023591-01, 1S10OD026833-01 and 1S10OD032169-01. The authors thank Jinghang Zhang, Fnu Aodengtuya and Ming Liu for assistance with flow cytometry, Robert Dubin and Xusheng Zhang for input on transcriptome analysis and data processing, and David Reynolds for providing technical expertise on scRNAseq methods. We also are grateful to colleagues who provided mouse lines used for this work, including Drs. Weiming Yuan and Peter Cresswell (hCD1dKI mice), and Mitchell Kronenberg (Jα18-/- mice). **Figure 1A** and Graphical abstract were created with BioRender.com (Albert Einstein College of Medicine license).

## Conflict of interest

The authors declare that the research was conducted in the absence of any commercial or financial relationships that could be construed as a potential conflict of interest.

## Publisher’s note

All claims expressed in this article are solely those of the authors and do not necessarily represent those of their affiliated organizations, or those of the publisher, the editors and the reviewers. Any product that may be evaluated in this article, or claim that may be made by its manufacturer, is not guaranteed or endorsed by the publisher.
