# Supplementary figures and images for: A humanized mouse model for in vivo evaluation of invariant Natural Killer T cell responses
Source: Front Immunol. 2022 Oct 3;13:1011209. doi: 10.3389/fimmu.2022.1011209 (PMC9574442; doi:10.3389/fimmu.2022.1011209)

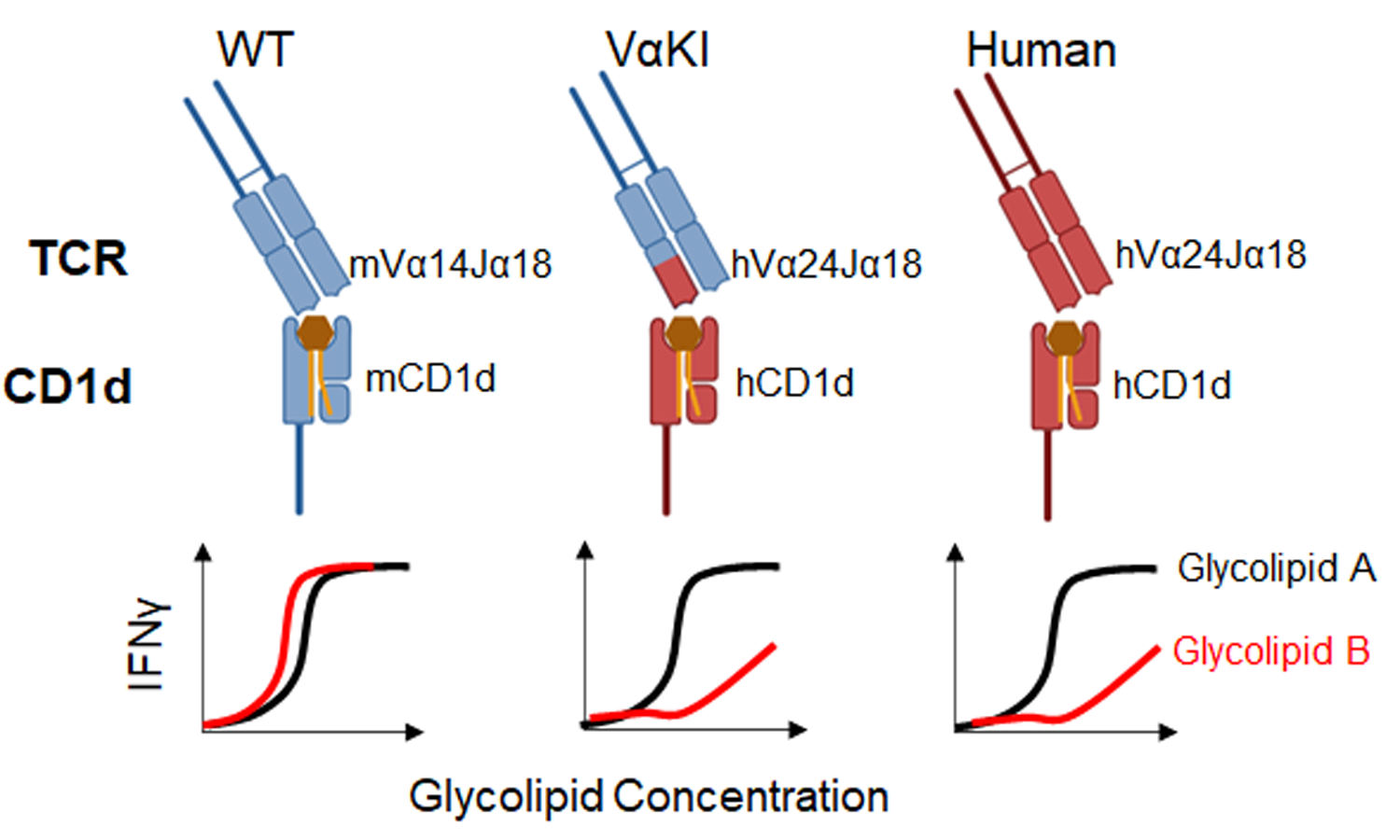

Supplement: Supplementary file 1 [file Image_1.tif]
