# Supplementary material for: A humanized mouse model for in vivo evaluation of invariant Natural Killer T cell responses
Source: Front Immunol. 2022 Oct 3;13:1011209. doi: 10.3389/fimmu.2022.1011209 (PMC9574442; doi:10.3389/fimmu.2022.1011209)
Supplement: Supplementary file 2 [file DataSheet_1.pdf]

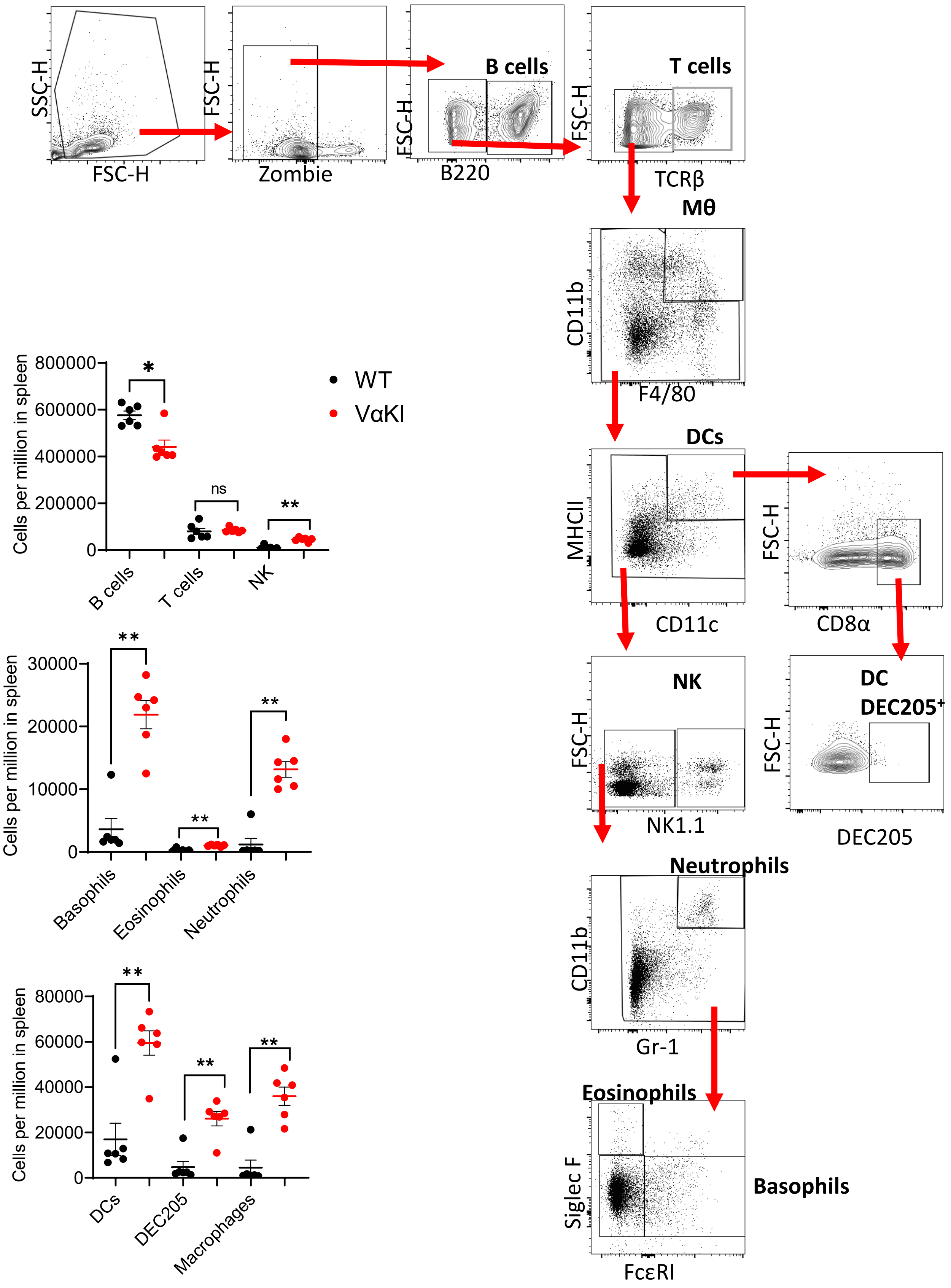

Supplementary Figure S1. Immune cells in C57BL/6 (WT) and VαKI mice. Gating strategy for leukocytes in spleen is shown, along with scatter plots of cell numbers from analysis of 6 WT and 6 VαKI mice. All pairwise comparisons shown were analyzed for significance using Mann-Whitney test. *P < 0.05, **P < 0.01.

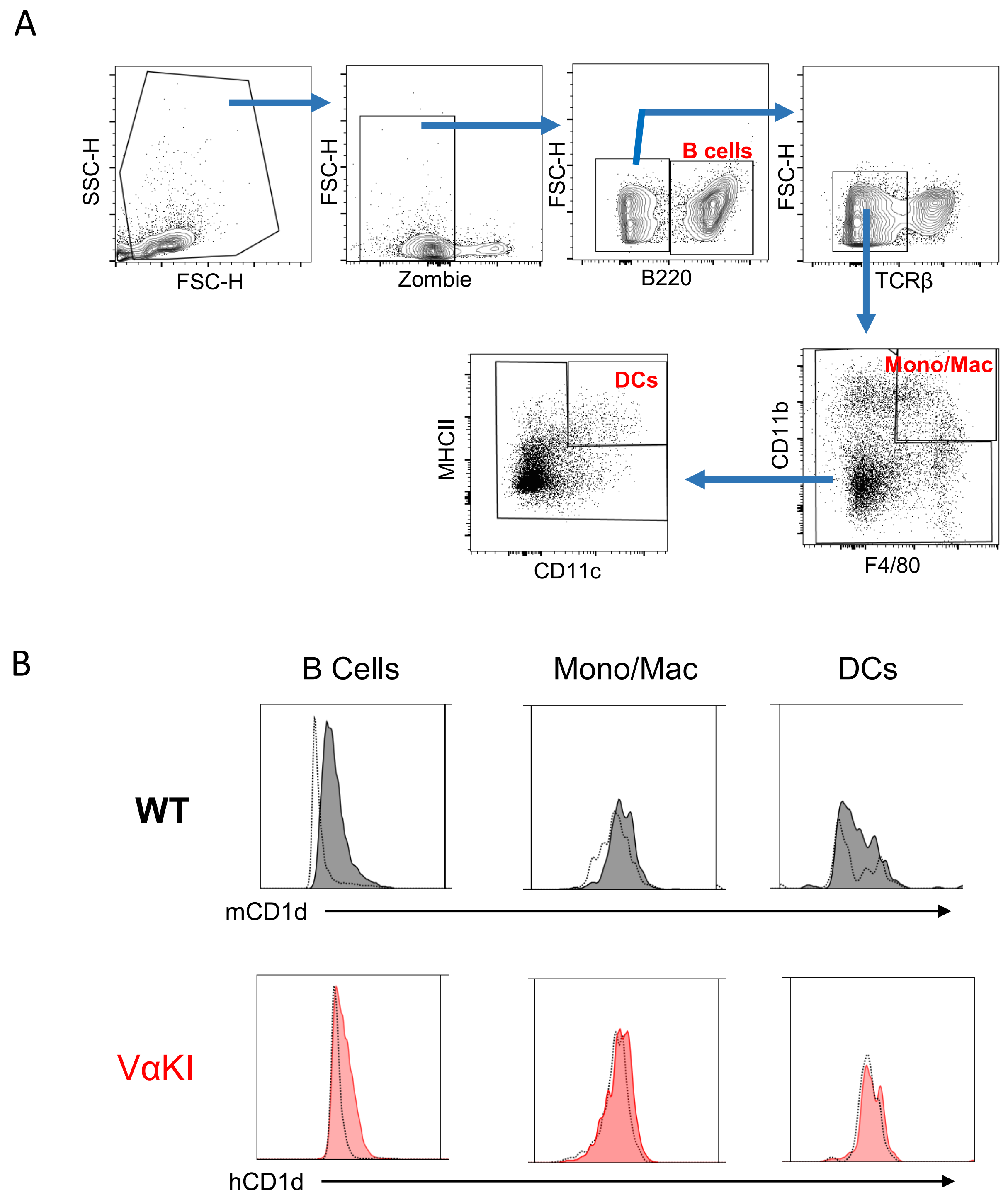

- **Supplementary Figure S2.** (A) Gating strategy to assess expression of mCD1d or hCD1d on B cells, monocytes/macrophages (Mo/Mac) and DCs in mouse PBMCs. (B) Filled histograms show mouse and human CD1d expression on indicated cells of WT (C57BL/6) or VαKI mice. The unshaded histograms are controls for background staining (identically stained cells from CD1d knockout mice).

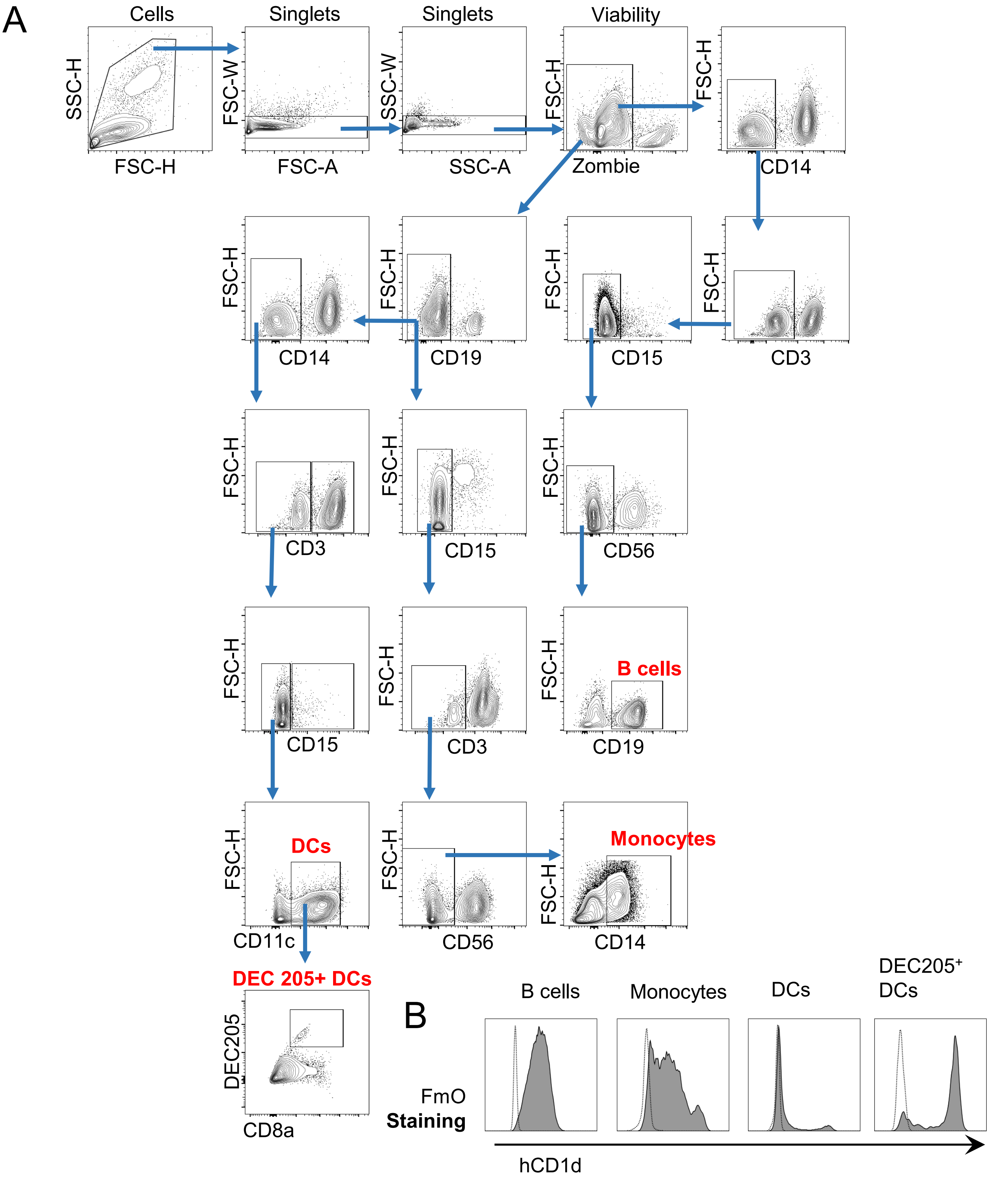

Supplementary Figure S3. FACS analysis of human CD1d expression on APCs from human PMBCs. Blood from random normal donors was separated by Ficoll gradient and stained for surface markers. (A) Live cells (Zombie negative) with exclusion of doublets were analyzed by FACS according to the gating scheme shown. (B) Human CD1d expression on the indicated cell types is shown as filled histograms. Open histograms are background staining based on standard fluorescence-minus-one (FmO) controls.

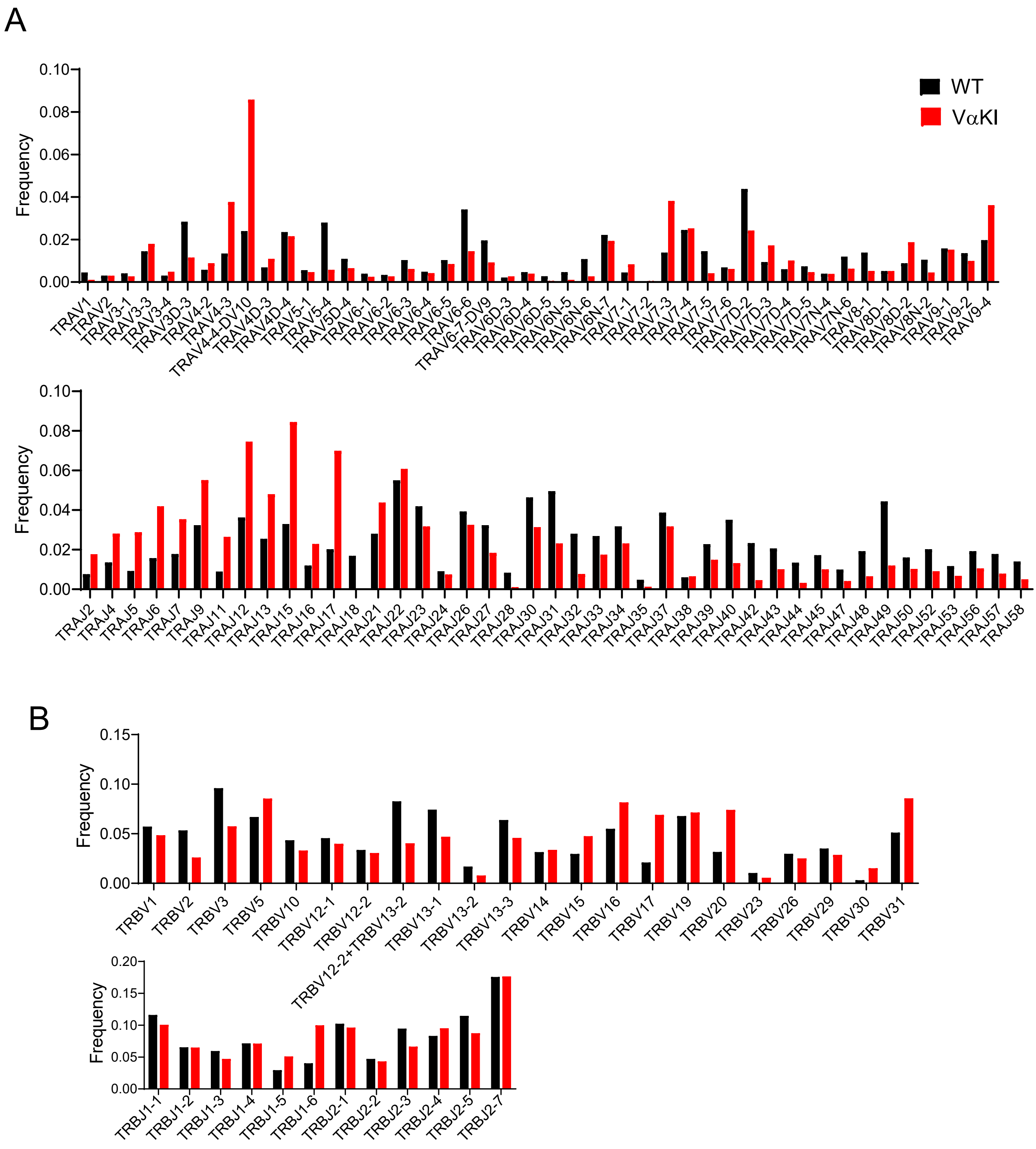

Supplemental Figure S4. Conventional MHC-restricted T cell TCR repertoire of VαKI mice shows normal diversity. V(D)J analysis of single-cell RNA sequencing of B220 negative, TCRβ^{+} and CD1d tetramer negative cells sorted from a pool of 3 spleens for each mouse strain. (A) Bar graphs frequencies of TCRα chain V and J genes and (B) of TCRβ chain V and J genes of WT and VαKI mice. Black bars are WT (C57BL/6) mice, and red bars are VαKI mice.

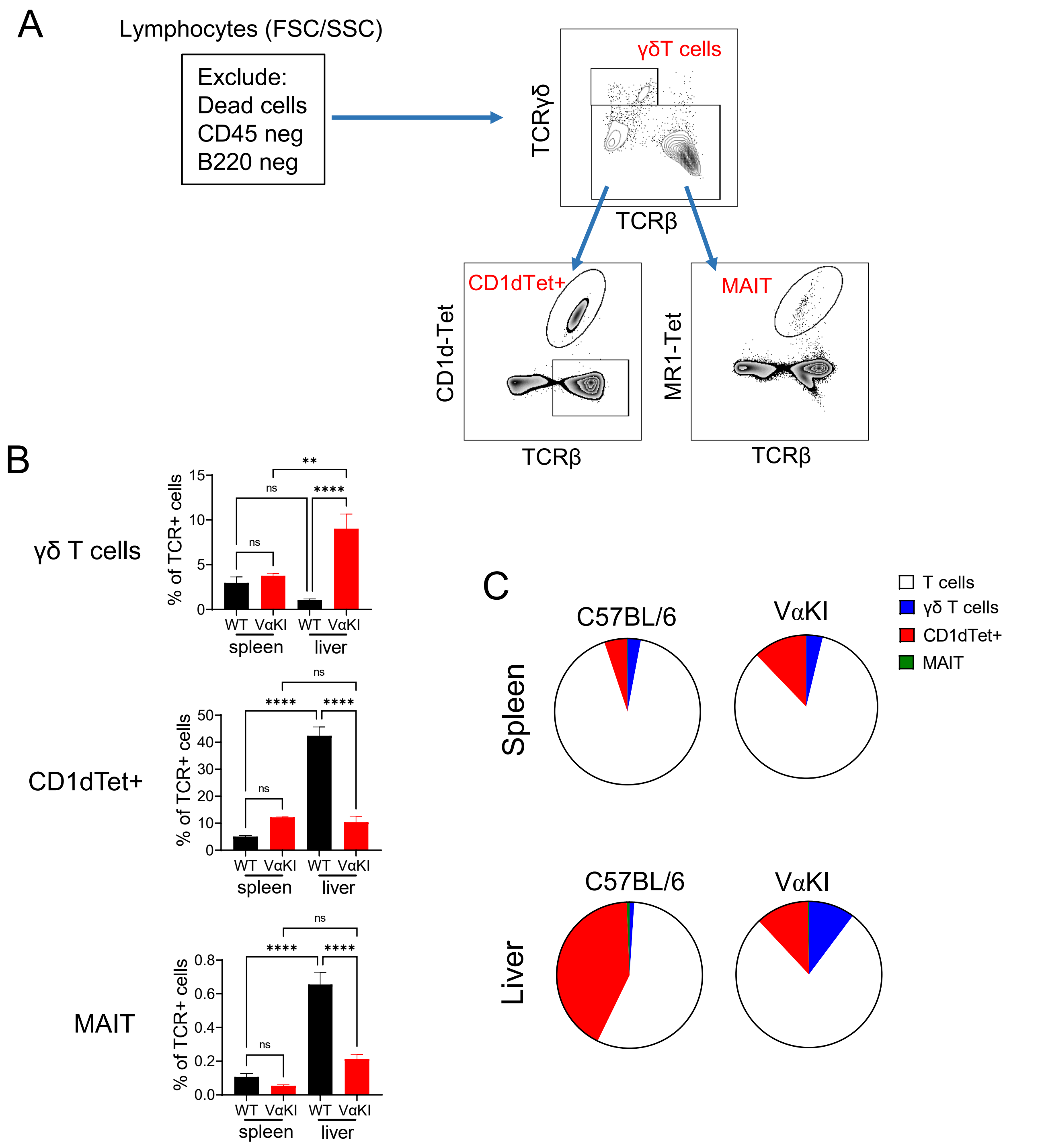

Supplemental Figure S5. Proportions of innate-like T cell subsets in VαKI mice. (A) Gating strategy used to differentiate γδ-T cells, CD1dTet+ cells and MAIT cells. (B) Bar graphs summarize percent of TCR positive (i.e., CD3+) cells for each subset for groups of 5 WT (C57BL/6) or VαKI mice. Means ± 1SE are shown. (C) Pie charts representing the data in B. Note that the green shaded slice representing MAIT cells is present but barely visible because of the extremely low numbers of these cells. All results are representative of two separate analyses. $^{**}P < 0.01$, $^{****}P < 0.0001$ (Mann-Whitney test).

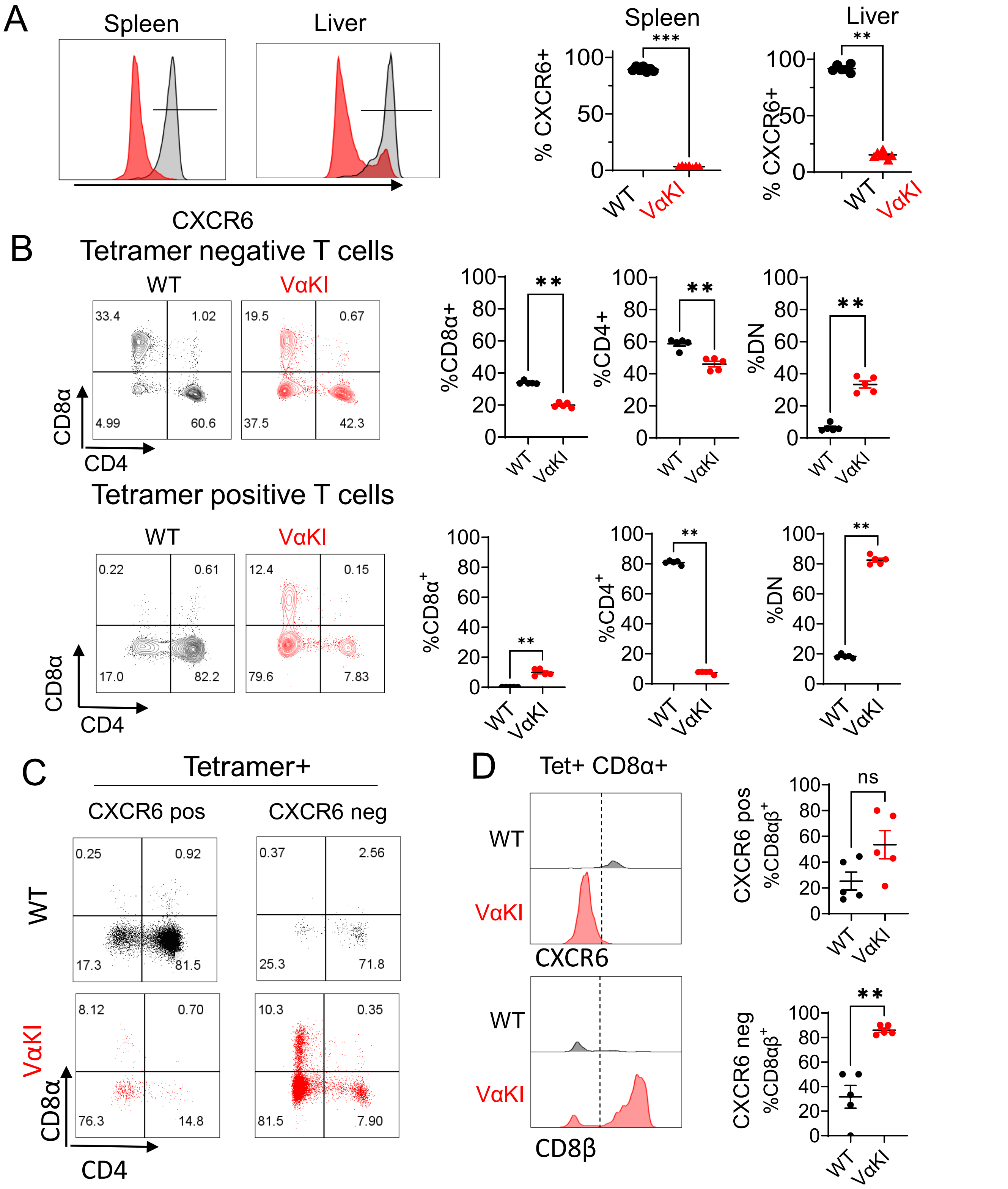

Supplementary Figure S6. CXCR6 and CD4/CD8 co-receptor expression by CD1d-tetramer binding cells in VαKI mice. (A) Histograms showing expression of CXCR6 on CD1d-Tet+ cells of WT (grey histograms) and VαKI (red histograms) mice from spleen and liver and their proportions in spleen and liver. Graphs to right summarize results for groups of 5 mice. Results shown are one of three separate experiments. (B) CD4 and CD8α proportions of tetramer negative and tetramer positive cells in WT (C57BL/6) and VαKI mice. Representative FACS plots shown are gated on TCRβB220 negative live lymphocytes. Graphs to right summarize results for groups of five mice. (C) CD4 and CD8α proportions of CD1d tetramer+ cells separated on basis of the expression of CXCR6. (D) Splenocytes gated for CD1d-tetramer+ and CD8α+ expression were analyzed for CXCR6 expression and CD8β expression. Representative histograms for one WT animal and one VαKI mouse are shown. Small size of histograms for the WT mouse reflects the low numbers of CD8α expressing cells compared with VαKI mice. Graphs to right summarize the expression of CD8αβ on the CXCR6 positive and negative subsets for groups of five mice. Means and 1SE are indicated. All pairwise comparisons shown were analyzed for significance using Mann-Whitney test. **P < 0.01, ***P < 0.001.

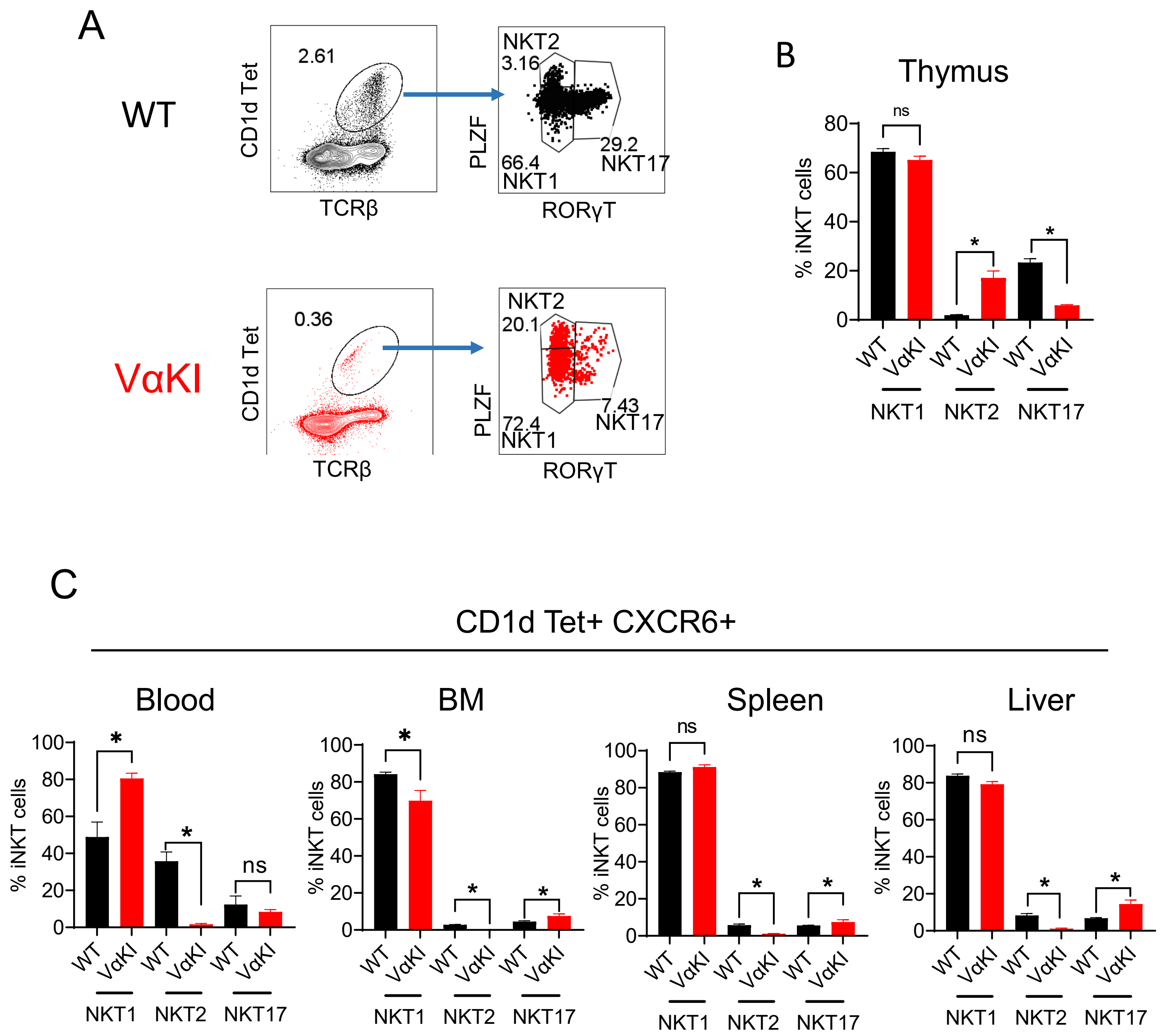

Supplementary Figure S7. FACS analysis demonstrating functional subsets of iNKT1, iNKT2 and iNKT17 cells. (A) Thymocytes of C57BL/6 (WT) and VαKI mice were stained for surface and intracellular markers including Zombie Live/Dead dye and mAbs specific for CD45, B220, TCRβ, PLZF, RORγT and either mouse (for WT) or human (for VαKI) CD1d tetramers loaded with glycolipid PBS57. Cells were gated for live, CD45+ B220 negative lymphocytes excluding doublets to generate the representative dot/contour plots shown. Numbers indicate percent of gated cells in each defined region. (B) Bar graph summarizing percent of CD1d Tet+ cells in each iNKT cell subset. (C) Bar graphs similar to (B) showing percent of subsets in cell suspensions of the indicated tissues. For extrathymic tissues, gating on CXCR6+ cells was included to exclude the major population of CXCR6 negative tetramer binding cells that lack features of canonical iNKT cells. All pairwise comparisons shown were analyzed for significance using Mann-Whitney test (*P < 0.05; ns, not significant).

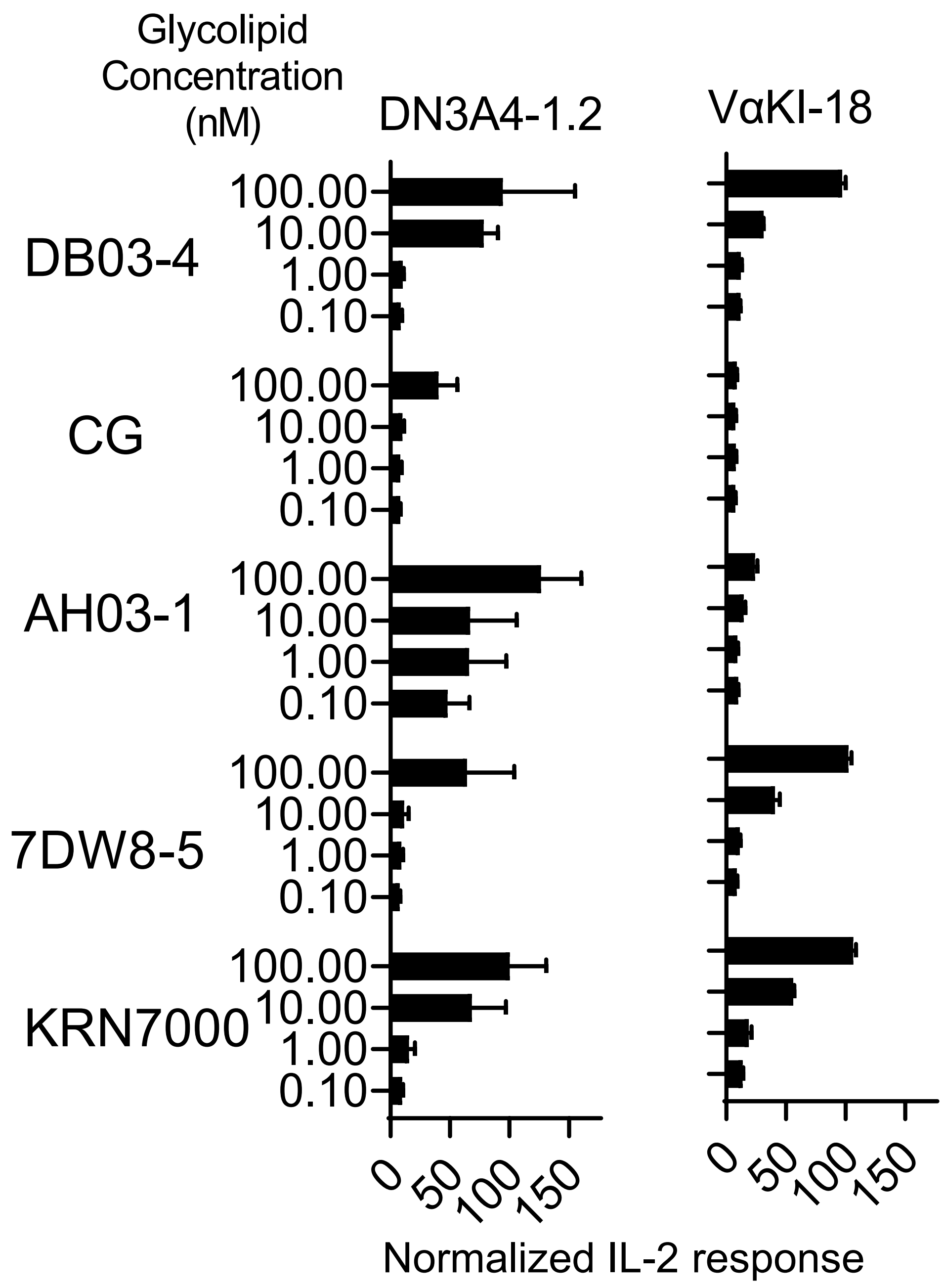

Supplementary Figure S8. Responses of cloned T cell hybridoma lines derived from WT mice (DN3A4-1.2) versus VαKI mice (VαKI-18) mice to αGalCer analogues. Hybridoma cells were stimulated with different concentrations of glycolipids presented by syngeneic BMDCs (i.e., C57BL/6 BMDCs for DN3A4-1.2 and VαKI BMDCs for VαKI-18., After 48h of culture, supernatant levels of IL-2 were determined by ELISA. Results were normalized to IL-2 level stimulated by 100 nM of KRN7000. Bars are mean ±1 SE for triplicate cultures. Results shown are representative of three separate experiments.
